# Efficacy, safety and exploratory analysis of neoadjuvant tislelizumab (a PD-1 inhibitor) plus nab-paclitaxel followed by epirubicin/cyclophosphamide for triple-negative breast cancer: a phase 2 TREND trial

**DOI:** 10.1038/s41392-025-02254-3

**Published:** 2025-05-26

**Authors:** Qiang Zhang, Mozhi Wang, Yumeng Li, Hengjun Zhang, Yusong Wang, Xiuyun Chen, Litong Yao, Mingke Cui, Haoran Dong, Xiang Li, Jian Liu, Bo Zhu, Yingying Xu

**Affiliations:** 1 Department of Breast Surgery, Liaoning Cancer Hospital and Institute, Shenyang, Liaoning 110801 P. R. China; 2 Department of Breast Surgery, The First Hospital of China Medical University, Shenyang, Liaoning 110001 P. R. China; 3 Department of Ultrasound, The First Hospital of China Medical University, Shenyang, Liaoning 110001 P. R. China; 4 Centre for Bioinformatics and Intelligent Medicine, Nankai University, Tianjin, 300071 P. R. China; 5 College of Computer Science, Nankai University, Tianjin, 300071 P. R. China; 6 Institute of Cancer, Xinqiao Hospital, Third Military Medical University, Chongqing, 400037 P. R. China

**Keywords:** Breast cancer, Tumour immunology

## Abstract

The optimal chemotherapy backbone and specific population of triple-negative breast cancer (TNBC) patients that benefit from neoadjuvant immunotherapy are not well established. This prospective, single-arm, phase II TREND trial assessed the efficacy and safety of tislelizumab plus nab-paclitaxel and epirubicin/cyclophosphamide-based chemotherapy as a neoadjuvant treatment for TNBC (ChiCTR2000035262). The primary endpoint was pathological complete response (pCR), with the secondary endpoints including safety assessment and objective response rate (ORR). ScRNA-seq, bulk RNA-seq, TCR-seq, cyTOF and WES were performed on pre-treatment and post-treatment samples. Among 53 total enrolled patients, 44 completed the combined neoadjuvant therapy, and 30 of 44 patients (68.18%) achieved pCR. Additionally, 14 out of 44 patients had a complete response (31.82%), with an ORR of 93.18%. The most commonly observed treatment-related adverse events (TRAEs) were alopecia, nausea and liver injury with 6 cases classified as grade 3 or higher adverse events. Immune response-related pathways, including TNF signaling pathway, T cell receptor signaling pathway, were enriched in pCR group. Pre-treatment model was identified and construct to predict response to immunotherapy. CDKN1A+ CD8 T lymphocytes were enriched in pCR group after neoadjuvant immunotherapy. Dynamic change of immune-related pathways at an early stage during the neoadjuvant immunotherapy may be associated with the treatment efficacy. In conclusion, neoadjuvant treatment of tislelizumab with nab-paclitaxel and anthracycline-based chemotherapy showed promising clinical activity and was well-tolerated among TNBC patients, without high incidence of TRAEs. These findings provide evidence supporting neoadjuvant tislelizumab with chemotherapy as an effective rational approach for treating TNBC.

## Introduction

Characterized by the absence of estrogen receptor, progesterone receptor, and epidermal growth factor receptor 2 (HER2) expression, triple-negative breast cancer (TNBC) is the most aggressive subtype with limited therapeutic targets beyond chemotherapy, leading to the highest rates of recurrence, metastasis, and poorest prognosis. Compared to other subtypes of breast cancer, TNBC exhibits higher tumor mutational burden (TMB), increased tumor-infiltrating lymphocytes (TILs), and elevated programmed cell death 1 ligand 1 (PD-L1) expression, suggesting enhanced immunogenicity and potential responsiveness to immune checkpoint inhibitors (ICI). IMpassion130 demonstrated that combining the PD-L1 inhibitor atezolizumab with nab-paclitaxel (nab-P) significantly improved progression-free survival (PFS) in PD-L1-positive patients with advanced TNBC.^1^ KEYNOTE-355 showed that pembrolizumab plus chemotherapy could prolong PFS and overall survival (OS) compared to chemotherapy alone in advanced TNBC patients with a combined positive score (CPS) ≥ 10.^2^

In early TNBC, the KEYNOTE-522 and IMpassion-031 trials demonstrated that neoadjuvant ICIs could increase pathologic complete response (pCR) rate and prolong event-free survival (EFS) compared to chemotherapy alone.^3–5^ Yet despite these encouraging results, the addition of ICI has shown no significant difference in benefit between subpopulations with different status of PD-L1 in neoadjuvant clinical trials for TNBC. TNBC is highly heterogeneous in clinical characteristics and treatment response, consequently presenting a challenge for therapeutic targeting and development of precise treatment.^6^ In order to identify the specific population that could potentially benefit from immunotherapy, further trials are needed to define the characteristics and novel biomarkers of immunotherapy response in TNBC.

Neoadjuvant immunotherapy exploits the relatively high tumor antigen load in TNBC patients, facilitating effector T cell activation to enhance the surveillance for and eradication of subclinical metastatic lesions, which has shown promise in early clinical trials.^7^ However, in a NeoTRIP trial, the addition of atezolizumab to neoadjuvant chemotherapy with carboplatin and nab-P for 8 cycles failed to reach a statistical increase in pCR rate and EFS.^8^ Though anthracycline- and taxane-based chemotherapy has been prioritized in the neoadjuvant chemotherapy for TNBC,^9^ the platinum-based chemotherapy is still commonly used as the backbone of neoadjuvant immunotherapy with increased toxicity.^10,11^ Therefore, the optimal chemotherapy backbone for immunotherapy is still under debate.

Tislelizumab, a humanized IgG4 variant monoclonal antibody targeting programmed cell death protein 1 (PD-1) with exceptional specificity, has shown higher affinity for PD-1 and a slower dissociation rate than pembrolizumab, which could theoretically improve efficacy.^12^ Notably, tislelizumab plus chemotherapy has been reported to improve survival as a first-line treatment for esophageal squamous cell carcinoma (ESCC) and metastatic nasopharyngeal cancer.^13,14^ However, few studies on its use in neoadjuvant therapy of TNBC have been reported.

Thus, we conducted a prospective, single-arm clinical study of **T**islelizumab in combination with nab-P followed by tislelizumab plus epi**r**ubicin/cyclophosphamid**e** (EC) in the **n**eoa**d**juvant treatment of TNBC (TREND, trial number: ChiCTR2000035262).^15^ Here, we evaluated response to platinum-free and low-dose chemotherapy combined with tislelizumab and identified crucial biomarkers, immune subgroups, and characteristics in the tumor microenvironment (TME) during neoadjuvant immunotherapy in TNBC. This study aimed to evaluate the efficacy and safety of tislelizumab combined with a platinum-free, low-dose chemotherapy backbone in TNBC neoadjuvant therapy, and identify predictive biomarkers and characterize dynamic immune microenvironment associated with response to neoadjuvant immunotherapy. Ultimately, we sought to define the population who would benefit from immunotherapy and provide more robust evidence for neoadjuvant immunotherapy and chemotherapy in TNBC.

## Results

### Patient characteristics of the TREND trial

**Fig. 1:**
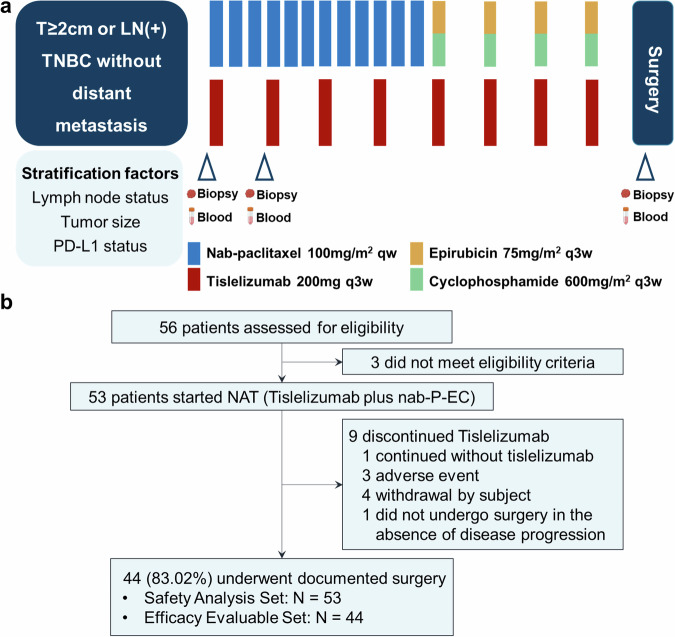
Trial design. **a** Study design for the TREND trial, **b** Flowchart for patient enrollment

From Nov 2020 through June 2023, a total of 56 patients diagnosed with TNBC were screened for eligibility. After excluding three patients who did not meet eligibility criteria, 53 patients were finally enrolled and received Tislelizumab plus nab-paclitaxel and anthracycline-based neoadjuvant treatment, and were included in the safety analysis set (SS, Fig. 1). By the data cutoff (June 30, 2023), the median follow-up time was 18.1 months (range: 4.8–31.9 months). Median age in the SS was 49 years old and 52.83% were premenopausal. Classification according to cancer stage identified 41 patients (77.36%) diagnosed as T2 stage, 31 patients (58.49%) with N1 stage, and 24 patients (45.28%) at the IIB stage, according to American Joint Committee on Cancer Staging Manual (AJCC, eighth edition).

Of the 53 participants, four withdrew consent and discontinued treatment, including four patients who refrained from scheduled treatment due to COVID-19 and one patient who discontinued treatment due to personally financial burdens. Another four patients discontinued neoadjuvant therapy following adverse events, three of whom discontinued without surgery and one who continued neoadjuvant chemotherapy (P-EC) without tislelizumab and accepted surgery. In addition, one patient completed the neoadjuvant therapy as allocated but did not undergo surgery in the absence of disease progression. Ultimately, 44 (83.02%) patients underwent surgery and were consequently included in efficacy evaluable set (EES). The clinical characteristics before neoadjuvant therapy of patients included in the SS and EES are shown in Supplementary Table S1.

### Efficacy

**Fig. 2:**
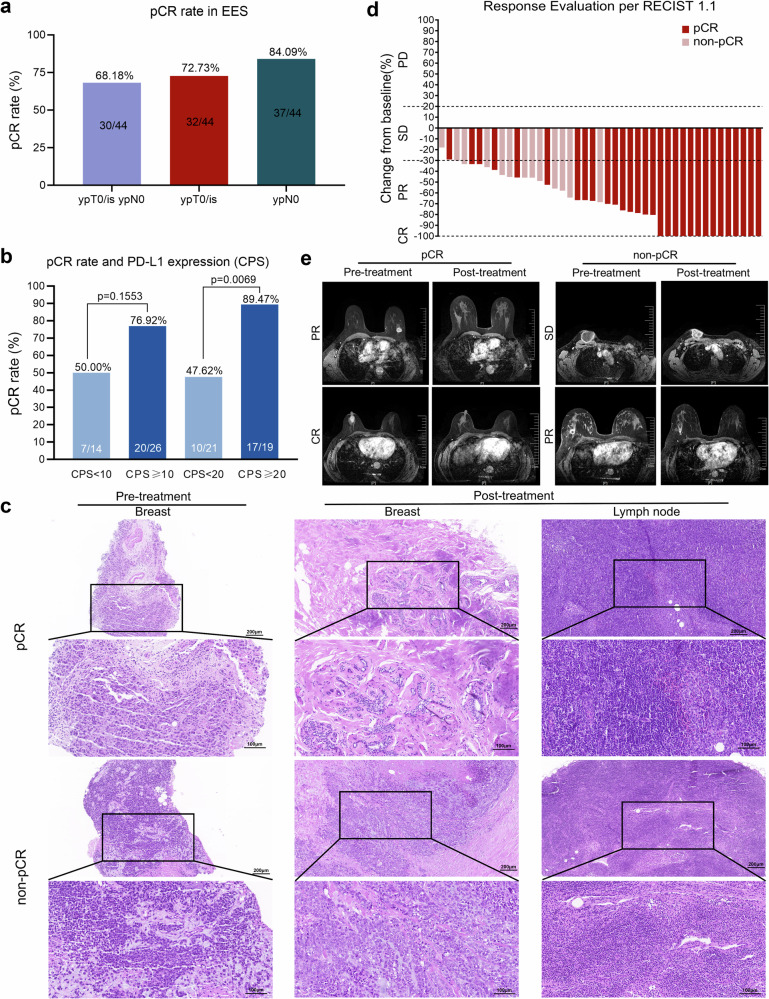
Efficacy of TREND trial in EES. **a** pCR rate in primary tumors (ypT0/is) and axillary lymph nodes (ypN0) after neoadjuvant treatment, **b** Relationship between tpCR rate and CPS (left, CPS threshold =10; right, CPS threshold = 20), **c** Representative images of H&E staining in one pCR patient (no.12) and one non-pCR patient (no.10) before (left) and after (right) neoadjuvant treatment, **d** Waterfall plots of the best clinical response in EES, **e** Magnetic resonance imaging of representative clinical responses in two pCR patients (no.24, no.47) and two non-pCR patients (no.10, no.13) before and after neoadjuvant treatment. pCR pathological complete response, EES efficacy evaluable set, RECIST Response Evaluation Criteria in Solid Tumors, CR complete response, PR partial response, SD stable disease, PD progressive disease, CPS combined positive score, H&E hematoxylin and eosin. Data cutoff: June 30, 2023

Primary analysis of pCR in the EES showed that 68.18% (30/44) achieved total pCR (tpCR, including ypT0/Tis ypN0). Further efficacy assessment specifically in primary tumors (ypT0/Tis) or axillary lymph nodes (ypN0) revealed that 72.73% (32/44) of patients achieved breast pCR (bpCR, ypT0/Tis), while ypN0 was attained in 84.09% (37/44) (Fig. 2a).

For subgroup analyses to define populations with positive response to neoadjuvant tislelizumab and chemotherapy, patients in EES were grouped by CPS for PD-L1 (Fig. 2b). At a cut-off value of 10, no significant difference in treatment response was found between the CPS-high and CPS-low groups. However, at a cut-off of 20, 89.47% (17/19) of CPS-high patients achieved tpCR, compared to 47.62% (10/21) in the CPS-low group (*p* = 0.0069). However, when grouped by T stage, N stage, AJCC stage, or tumor positive scores (TPS) of PD-L1, tpCR rates showed no differences between groups (Supplementary Fig. S1). These results suggested that high CPS (i.e., >20) could potentially serve as a biomarker for predicting neoadjuvant immune therapy efficacy in early TNBC (eTNBC) patients. Pathological responses before and after neoadjuvant treatment in two representative cases are presented in Fig. 2c.

Evaluation of the secondary endpoint, objective response rate (ORR), in the EES using Response Evaluation Criteria in Solid Tumors (RECIST) V1.1 definitions (Supplementary Table S2) showed that 61.36% (27/44) of patients reached partial response (PR), while 31.82% (14/44) reached complete response (CR), resulting in an ORR of 93.18% and a stable disease (SD) rate of 4.55% (2/44). Overall, the favorable disease control rate (DCR = CR + PR + SD) was observed as 97.73% (43/44). The best overall changes from pre-neoadjuvant to post-neoadjuvant (at surgery) for all evaluable patients in the EES are shown in Fig. 2d. Representative cases before and after neoadjuvant treatment are shown in Fig. 2e.

### Safety and feasibility

Assessment of safety and toxicity, including all-grade treatment-related adverse events (TRAEs), for patients in the SS are summarized Table 1. Overall, 90.57% (48/53) patients experienced any grade of TRAE, 11.32% (6/53) of whom experienced a grade 3 or 4 TRAE. Among TRAE types, 62.26% (33/53) presented with alopecia, 50.94% (27/53) experienced nausea, 33.96% (18/53) had liver injury, 33.96% (18/53) with anemia, 17 (32.08%) with fatigue, 16 (30.19%) with vomit, 22.64% (12/53) with neutropenia, 20.75% (11/53) with leucopenia, 1 (1.89%) with cough, 26.42% (14/53) presented with rash, and 1 (1.89%) with diarrhea. Hypothyroidism (24.53%, 13/53) was the most frequent immune-related adverse events, followed by hyperthyroidism (16.98%, 9/53), vitiligo (2, 3.77%) and immune-related pneumonia (2, 3.77%). Among severe TRAEs, 3 (5.66%) patients had grade 3-4 liver injury with increased AST/ALT, 2 (3.77%) had grade 3-4 neutropenia, 1 (1.89%) had grade 3-4 leucopenia, and 1 (1.89%) had vitiligo. Ultimately, 4 (7.55%) patients discontinued neoadjuvant therapy due to TRAEs.

**Table 1:** Treatment-related adverse events during neoadjuvant treatment in SS

Treatment-related adverse events	Grades, *n* (%)	
	Any grades	Grade 3–4
Treatment-related adverse events		
Alopecia	33 (62.26%)	0 (0.00%)
Nausea	27 (50.94%)	0 (0.00%)
Liver injury (AST/ALT increased)	18 (33.96%)	3 (5.66%)
Anemia	18 (33.96%)	0 (0.00%)
Fatigue	17 (32.08%)	0 (0.00%)
Vomit	16 (30.19%)	0 (0.00%)
Rash	14 (26.42%)	0 (0.00%)
Neutropenia	12 (22.64%)	2 (3.77%)
Leucopenia	11 (20.75%)	1 (1.89%)
Cough	1 (1.89%)	0 (0.00%)
Colitis	1 (1.89%)	0 (0.00%)
Adverse event of interest		
Hypothyroidism	13 (24.53%)	0 (0.00%)
Hyperthyroidism	9 (16.98%)	0 (0.00%)
Vitiligo	2 (3.77%)	1 (1.89%)
Immune-related pneumonia	2 (3.77%)	0 (0.00%)

SS, safety analysis set

### Genomic landscape

Tumor tissues and matched normal tissues from 16 patients (11 pCR patients and 5 non-pCR patients) were collected for tumor mutation load via whole exome sequencing (WES). The most frequent mutation type was missense mutation and frequently altered genes were *WNK2* (18.75%) and *PRKAR1A* (18.75%) (Supplementary Fig. S2a, b). *PRKAR1A*, *ALP* and *ADGRB2* mutations occurred in 27.3% (3/11) of the pre-neoadjuvant pCR samples while in 0 of non-pCR samples (0/5), possibly indicating a trend of good response (Supplementary Fig. S2a). However, no significant differences were presented between pCR and non-pCR patients in TMB (Supplementary Fig. S2c, d).

### Gene expression profile before neoadjuvant therapy

To better understand the differences between pCR and non-pCR patients and dig out potential biomarkers of response to neoadjuvant immunotherapy, we collected biopsy samples, peripheral blood and tissues resected in the surgery before neoadjuvant (C0), after 1 cycle of neoadjuvant therapy (C1) and after 8 cycles of neoadjuvant (at surgery, S) from patients in TREND trial and performed bulk RNA-seq, single-cell RNA-seq (scRNA-seq), T cell receptors (TCR)-seq, mass cytometry (cytometry by time-of-flight, CyTOF) and WES.

**Fig. 3:**
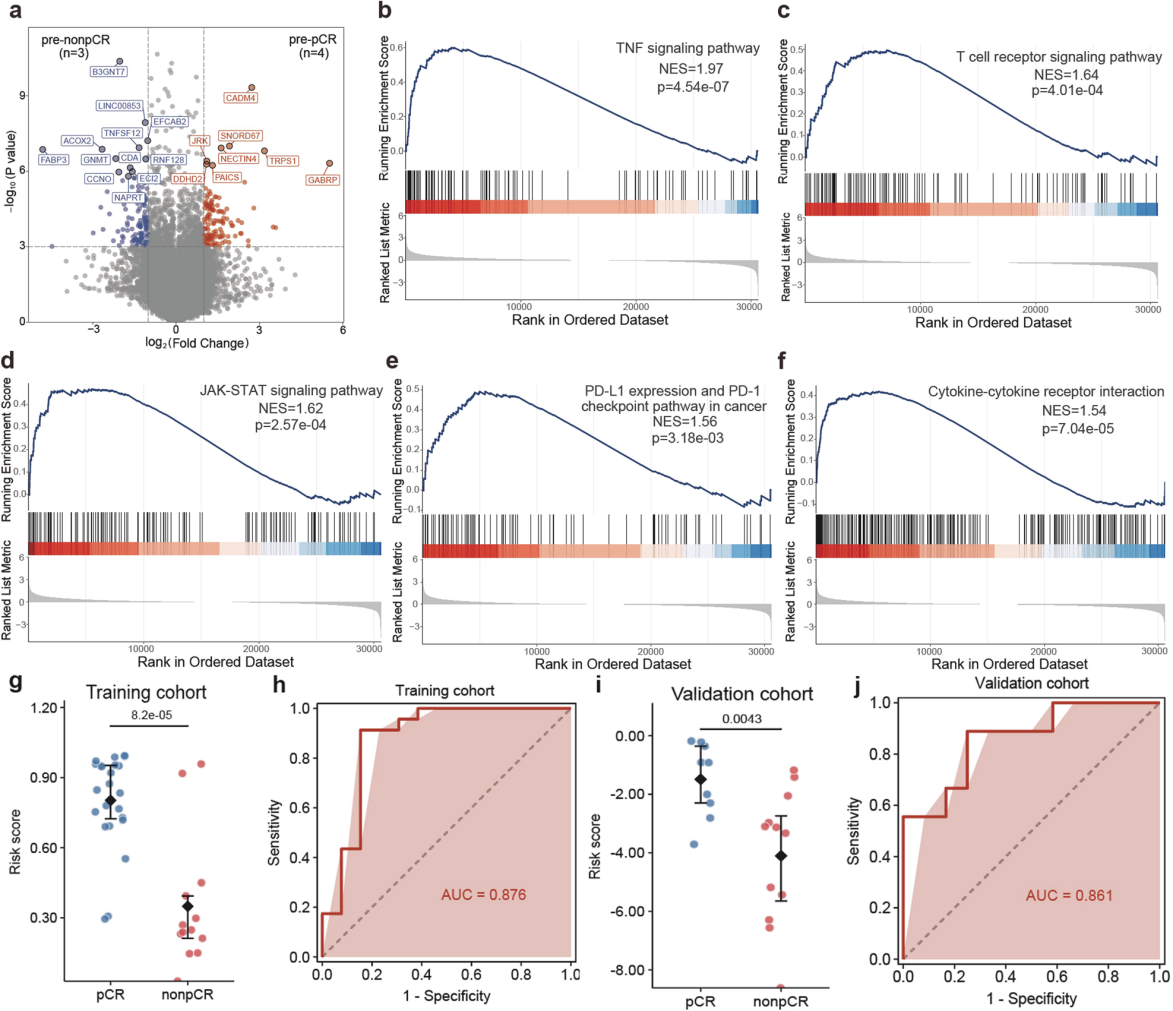
Gene expression profile before neoadjuvant therapy and predictive index of response. **a** The volcano plot of transcriptomic characteristics between pCR and non-pCR patients at baseline (pre-neoadjuvant), **b**–**f** Gene set enrichment analysis of the Hallmark TNF, T cell receptor and JAK-STAT signaling pathway, PD-L1/PD-1 checkpoint pathway in cancers and cytokine-cytokine receptor interaction, **g** Box plot of the predictive score between pCR and non-pCR patients in training cohort, **h** The ROC curve of the predictive model in training cohort, **i** Box plot of the predictive score between pCR and non-pCR patients in validation cohort, **j** The ROC curve of the predictive model in training cohort. pCR pathological complete response, TNF tumor necrosis factor, NES normalized enrichment score, JAK Janus Kinase, STAT signal transducer and activator of transcription, PD-L1 programmed cell death 1 ligand 1, PD-1 programmed cell death-1, AUC area under the curve, ROC curve receiver operating characteristic curve

To profile the transcriptional differences before neoadjuvant therapy between pCR and non-pCR patients, we performed differential expression gene (DEG) analysis on pre-neoadjuvant samples (Fig. 3a). Genes on lipid metabolism (*FABP3* and *ACOX2*) were increased in non-pCR group, while genes including *GABRP*, *NECTIN4* and *CADM4* were found elevated in pCR group before neoadjuvant therapy. Subsequent gene set enrichment analysis (GSEA) based on DEGs demonstrated that immune response-related pathways were enriched in pCR group, including TNF signaling pathway, T cell receptor signaling pathway, JAK-STAT signaling pathway, PD-L1 expression and PD-1 checkpoint pathway in cancers and cytokine-cytokine receptor interaction (Fig. 3b-f).

As shown in Fig. 3g, we identified 3 DEGs between pCR and non-pCR patients from samples at C0 from TREND and TNBC patients received neoadjuvant pembrolizumab and chemotherapy from I-SPY2 trial. Above gene set was trained by generalized linear model to construct a signature to define the characteristics of pCR and non-pCR patients and predict the efficacy of neoadjuvant immunotherapy and chemotherapy. The final model showed a significant difference between pCR and non-pCR patients in our test cohort with an area under the curve (AUC) of 0.876 (Fig. 3g, h, *p* < 0.001). To validate the accuracy of above model, we performed the analysis on a validation cohort and found that efficacy of neoadjuvant immunotherapy can be well distinguished with an AUC of 0.861 (Fig. 3i, j, *p* = 0.004).

### Dynamic change of TME

**Fig. 4:**
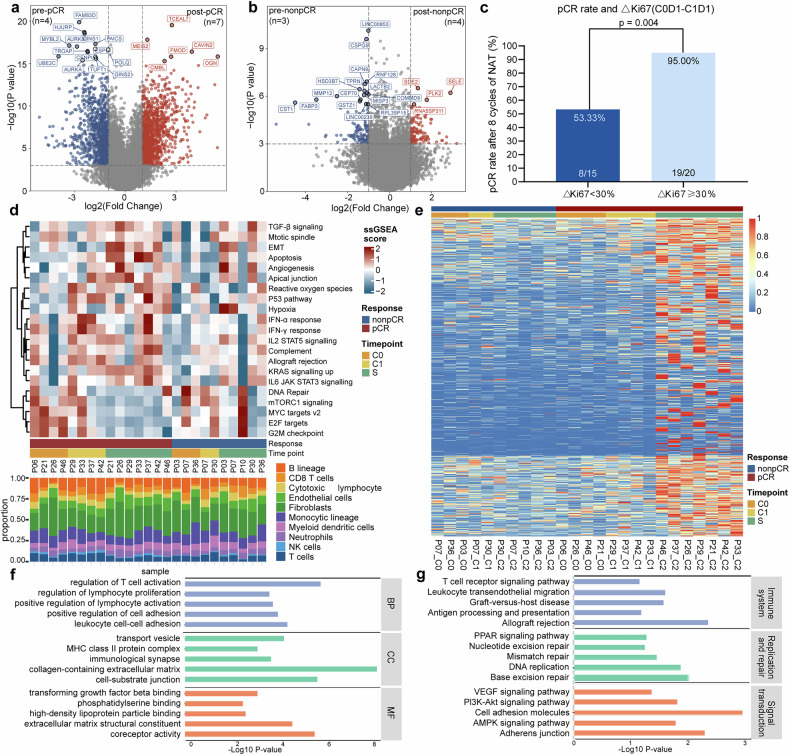
Dynamic change of TME during neoadjuvant tislelizumab and chemotherapy. **a** The volcano plot of transcriptomic characteristics between before and after neoadjuvant therapy in pCR patients, **b** The volcano plot of transcriptomic characteristics between before and after neoadjuvant therapy in non-pCR patients, **c** The relationship of pCR rate and dynamic change of ki67 from baseline, △ki67= ki67 at baseline to C1D1(Day1 of Cycle1), **d** ssGSEA analysis of RNA-seq data from tumor samples at baseline (C0), C1D1 and surgery (S), **e** Heatmap of genes in cluster 5, **f**, **g** GO and KEGG enrichment analysis of genes in cluster 5. pCR pathological complete response, NAT neoadjuvant therapy, C0D1 Day1 of Cycle0 (C0, baseline), C1D1 Day1 of Cycle1, GSVA gene set variation analysis, ssGSEA single sample gene set enrichment analysis, GO gene ontology, BP biological process, CC cellular component, MF molecular function, KEGG Kyoto Encyclopedia of Genes and Genomes

In addition to the DEGs before neoadjuvant therapy, we also compared the samples before and after neoadjuvant therapy in pCR and non-pCR patients. Analysis of DEGs showed obvious changes in transcripts of pCR patients with 1160 up-regulated genes and 1007 down-regulated genes (Fig. 4a). On the contrary, little changes in transcripts of non-pCR patients was observed with 112 up-regulated genes and 131 down-regulated genes (Fig. 4b). Above results demonstrated that dynamic change could describe the characteristics differences between pCR and non-pCR patients in enriched dimensions.

Thus, we included samples after 1 cycle of neoadjuvant therapy (C1) as well as pre- and post- neoadjuvant therapy (C0 and S) and analyzed the relationship of gene expression and clinical response. Interestingly, when the decrease of Ki-67 from C0 to C1 was more than 30%, 95% patients (19/20) tended to achieve pCR, which was significantly more than those whose decrease of Ki-67 was less than 30% (53.33%, *p* = 0.004, Fig. 4c). Gene set variation analysis (GSVA) at different time-point was also performed according to HALLMARK pathways in the Molecular Signatures database (Supplementary Fig. S3a-c). Based on above pathways enriched by GSVA, we next performed single sample gene set enrichment analysis (ssGSEA) and discovered that the immune-related pathways were significantly activated while the pathways about cell proliferation were inhibited after treatment in pCR patients (Fig. 4d). Importantly, in pCR patients, immune-related pathways enriched early at C1, which was exactly similar to that at surgery. In non-pCR patients, however, immune-related pathways enriched late and the enrichment of pathway at C1 was more similar to that at C0. These results demonstrated that massive disease regression and immune activation occurred at an early stage in pCR patients.

To profile the dynamic change during neoadjuvant therapy in detail, we performed k-means clustering based on spline fitting and assigned genes with similar characteristics to eight gene clusters and selected cluster 5 for downstream enrichment analysis (Supplementary Fig. S3d, Fig. 4e). Based on the clustering of fuzzy c-means algorithm, genes with similar features in pCR and non-pCR patients were also assigned to nine gene clusters respectively, and cluster 1 and 3 in pCR group and cluster 2 and 7 in non-pCR group were selected for next analysis (Supplementary Fig. S3e, f). Above gene clusters extracted by two algorithms were pooled and enriched. GO and KEGG analysis showed that T cell activation, lymphocyte proliferation and related immune function were activated in patients achieved pCR (Fig. 4f, g).

### Transcriptional and clonal diversity of CD8 T lymphocytes between pCR and non-pCR patients

**Fig. 5:**
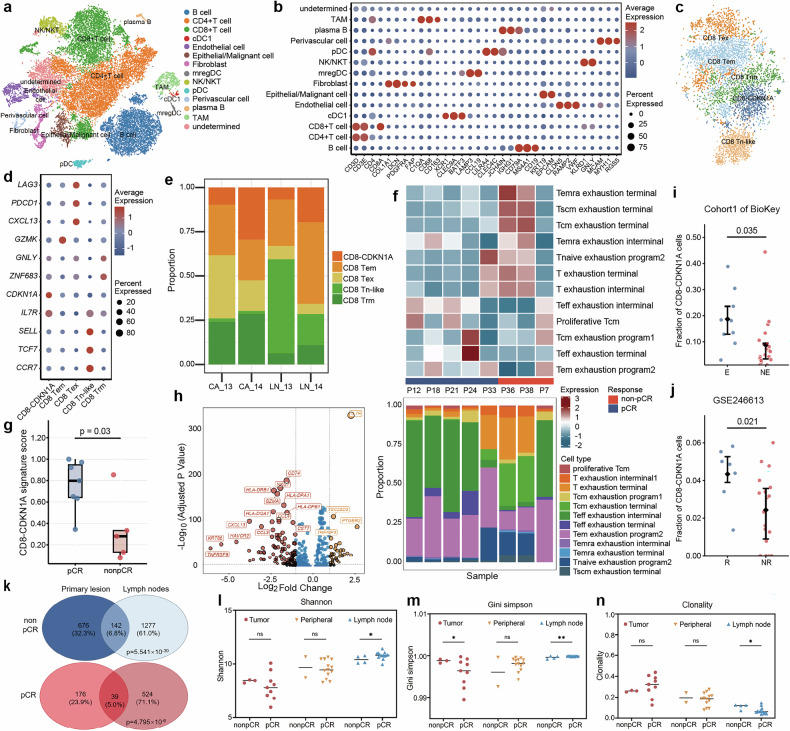
Transcriptional and clonal diversity of CD8 T lymphocytes between pCR and non-pCR patients. **a** tSNE of cells from total samples colored by cell type based on scRNA-seq, **b** Expressions of selected canonical marker genes in each major cell population based on scRNA-seq, **c** tSNE of CD8 T lymphocytes from total samples colored by cell type based on scRNA-seq, **d** Expressions of selected canonical marker genes in each major CD8 T lymphocytes populations based on scRNA-seq, **e** abundance of major CD8 T lymphocytes populations in each tissue sample based on scRNA-seq, **f** Heatmap and abundance of major CD8 T lymphocytes populations in all primary tumors based on cyTOF data, **g** validation of CDKN1A+ CD8 T lymphocytes score in pCR and non-pCR patients based on RNA-seq data. **h** Volcano plot showing differentially expressed genes between CDKN1A+ CD8 T lymphocytes and CDKN1A- CD8 T lymphocytes, **i** External validation of CDKN1A+ CD8 T lymphocytes in BioKey, **j** External validation of CDKN1A+ CD8 T lymphocytes in GSE246613, **k** Venn plot of the shared TCR clonetypes of CD8 T lymphocyte between primary lesions and lymph nodes, **l** Shannon Index of TCR in tumor, peripheral blood and lymph nodes between pCR and non-pCR patients, **m** Gini Simpson index of TCR in tumor, peripheral blood and lymph nodes between pCR and non-pCR patients, **n** TCR clonality in tumor, peripheral blood and lymph nodes between pCR and non-pCR patients. tSNE t-Distributed Stochastic Neighbor Embedding, pCR pathological complete response. E, T cell expansion. NE, no T cell expansion

Recent studies have shown that the efficacy of immune therapy would be affected by the tumor microenvironment, encompassing its cellular components and their states respectively.^16–18^ To test this possibility, we characterized differences in the TME between pCR and non-pCR patients based on scRNA-seq and paired scTCR-seq information from two patients and cyTOF data from another seven patients. After filtering for quality, we obtained 28,709 high-quality single-cell transcriptomes spanning 14 distinct cell populations, such as CD8 T lymphocytes, CD4 T lymphocytes, and B lymphocytes (Fig. 5a, b, Supplementary Fig. S4a). An enrichment of CD8 T lymphocytes and macrophages in non-pCR patients and fibroblast in pCR patients after combination therapy of ICI and chemotherapy can be observed in scRNA-seq and cyTOF analysis of primary lesion (Supplementary Fig. S4a, b). In lymph nodes that both reached pCR, B lymphocytes and macrophages were highly enriched in pCR patients distinctively (Supplementary Fig. S4c).

Infiltration levels of CD8 T lymphocytes have been considered a crucial determinant of ICI therapy efficacy. In non-pCR group, higher enrichment of CD8 T lymphocytes were observed. Subsequent analysis revealed that exhausted CD8 T lymphocytes (Tex), especially terminal Tex expanded in non-pCR group (Fig. 5c-f, Supplementary Fig. S4c, d). Interestingly, we identified a subpopulation of CD8 T lymphocytes that highly expressed *CDKN1A*, named as CDKN1A+ CD8 T lymphocytes, which was highly enriched in primary lesion and lymph nodes of pCR group (Fig. 5c-e, Supplementary Fig. S4e). To validate the significance of CDKN1A+ cells in response to ICI, we constructed a therapeutic index, CDKN1A+ CD8 T lymphocytes score and analyzed the relationship of CDKN1A score and response to neoadjuvant immunotherapy based on bulk-RNA-seq from 12 patients. The result showed that higher CDKN1A+ CD8 T lymphocytes was enriched in pCR group, suggesting that neoadjuvant tislelizumab may increase the immune response by elevated CDKN1A + CD8 T lymphocytes (*p* = 0.03, Fig. 5g). Consistently, external validation performed on another independent cohorts^19,20^ demonstrated significant enrichment of CDKN1A+ CD8  T lymphocytes in patients exhibiting enhanced response to immunotherapy (Fig. 5i,j, Supplementary Fig. S4b). Subsequently, DEG analysis showed that effector-related genes (*GZMB*, *GZMA*), exhaustion-related genes (*HAVCR2, TOX, TIGIT*), and HLA-related genes decreased, while the naïve and memory-related genes (*IL7R, GPR183*) enriched in CDKN1A+ CD8 T lymphocytes (Fig. 5h). These results demonstrated that elevated CDKN1A+ CD8 T lymphocytes with higher stemness and characteristics of memory cells may play a key role in response to neoadjuvant immunotherapy and chemotherapy.

To assess the differences of clonal relationship between CD8 T lymphocytes in metastatic lymph nodes and primary tumors, we identified those shared TCR clones between metastatic lymph nodes and primary tumors within each patient, and compared state changes of T cells from the same clones through a Relative Immunotherapeutic Effect Testing for Paired Samples (RIETPS). Similar clones were shared in CD8 T lymphocytes between primary tumors and metastatic lymph nodes of non-pCR and pCR patients (non-pCR: *p* = 5.541×10-30; pCR: *p* = 4.796×10-6; hypergeometric testing) (Fig. 5k, Supplementary Fig. S4f). However, enhanced TCR diversity and decreased TCR clonality can be observed in lymph nodes of pCR patients compared to non-pCR patients, whereas no significant differences in samples of primay tumor and peripheral blood (Fig. 5l-n, Supplementary Fig. S4g). Above observation indicated that enhanced TCR diversity in lymph nodes might fuel response to ICI instead of TCR clone sharing, making it possible to achieve disease control both during and after neoadjuvant therapy.

## Discussion

Pembrolizumab plus chemotherapy (*e.g*., carboplatin followed by EC) has been approved as a neoadjuvant treatment for TNBC based on the success of KEYNOTE-522.^4^ However, the optimal chemo-backbone and affected population most likely to benefit from neoadjuvant immunotherapy both remain undefined. Previous clinical trials that yielded positive results have predominantly focused on platinum-based (carboplatin in KEYNOTE-522 and NeoTRIPaPDL1) and dose-dense chemo-backbones (nab-P at 125 mg/m^2^ in IMpassion-031, NeoTRIPaPDL1, and GeparNuevo).^5,8,21^ However, platinum-based and dose-dense chemotherapy-induced adverse events, particularly severe myelosuppression, lead to prolonged inter-cycle intervals, ultimately diminishing the expected efficacy of neoadjuvant therapy. Here, in this current study, we conducted the first clinical trial assessing the safety and efficacy of tislelizumab in combination with nab-P-EC as a neoadjuvant therapy for TNBC in the previously established TREND cohort. This study reported favorable efficacy (pCR rate of 68.18%) and controllable toxicity for tislelizumab with a de-escalated platinum-free (nab-P-EC) and low-dose (nab-P at 100 mg/m^2^ and epirubicin at 75 mg/m^2^) chemo-backbone as a neoadjuvant therapy for achieving pCR and clinical response in TNBC. The initial rationale for combining chemotherapy with immunotherapy was to remodel the relatively low immunogenicity TME of breast cancer, thereby sensitizing the immunotherapy. Consistently, in the TONIC trial of metastatic TNBC, a 2-week induction cycle of doxorubicin before administering the PD-1 inhibitor, Nivolumab, resulted in a higher ORR (35%) than non-induction (17%), as well as induction with cyclophosphamide (8%) or cisplatin (23%).^22^ As a strong cytotoxic antibiotic, epirubicin exerts both a direct killing effect and indirect induction of immunogenic death on tumor cells, which activating antigen presentation in dendritic cells and enhancing priming of CD8 T lymphocytes.^23^ Our results suggested that platinum-free and low-dose epirubicin can provide benefit without uncontrollable TRAEs, demonstrating the favorable compatibility of anthracyclines and immunotherapy. Additionally, percentage of population at T1/T2 in TREND trial are highly 83%, while 74% in KEYNOTE-522 and 55% in NeoTRIPaPDL1. Patients with relatively low tumor burden may benefit a lot from immunotherapy due to the intact immune status compared to that of locally advanced patients. Notably, this study provides evidence for future strategies that prioritize immunotherapy with moderate chemo-backbone as the neoadjuvant therapy for TNBC at early stage.

Identifying the most reliable methodology to evaluate immunotherapy efficacy remains a critical unmet need in the field. Interestingly, though all 14 patients who were assessed as CR subsequently achieved pCR, the imaging-based clinical response and pathology-based response in patients evaluated as PR were inconsistent. Among the 27 patients assessed as PR, 15 (55.56%) subsequently achieved pCR, while 12 (44.44%) had residual invasive disease. This discrepancy highlights the limitations of response evaluation criteria based on imaging in capturing the full extent of pathologic tumor regression following neoadjuvant immunotherapy, where immune-related pseudoprogression or delayed immune activation may obscure true pathologic responses. Current response evaluation criteria for immunotherapy in advanced solid tumors remain predominantly based on imaging, yet pathological diagnosis retains superior accuracy. Thus, a more accurate evaluation criteria tailored to immunotherapy efficacy remains to be established. In this context, the neoadjuvant cohort holds potential as a feasible platform for exploring methodologies and validating refined evaluation criteria for immunotherapy. Since long-term survival is the critical endpoint in TNBC, its correlation with the high ORR in this study remains uncertain. KEYNOTE-522 have revealed the durable anti-tumor effect of pembrolizumab. Although early pCR rates are encouraging in this trial, long-term follow-up ( ≥ 3 years) is ongoing to assess whether the observed response translates into durable EFS or OS benefits.

Combination of immunotherapy and chemotherapy is well-established to improve survival in PD-L1-positive metastatic TNBC patients,^1,2^ but may benefit all eTNBC patients, regardless of PD-L1 status. Although multivariate analysis showed that PD-L1 status was the most important factor affecting pCR rate in the NeoTRIP trial, subgroup analysis of NeoTRIP and IMpassion-031 results both failed to prove that the addition of ICI led to improved benefit in PD-L1-positive disease.^5,8^ The role and threshold of CPS in neoadjuvant immunotherapy remains controversial. Consistent with prior studies in NSCLC and gastric cancer, our results showed that tpCR rate was higher in the CPS-high (CPS ≥ 20) subgroup compared to the CPS-low (CPS < 20) subgroup, thus providing confirmatory evidence of the value of CPS for predicting efficacy and supporting its further validation through large-scale trials. CPS thresholds in this study were selected according to KEYNOTE-522, with comparable pCR rates observed in both CPS ≥ 20 and CPS ≥ 10 subgroups. However, the higher proportion of CPS ≥ 20 subgroup (19/44) than that of KEYNOTE-522 (126/401) likely contributed disproportionately to the overall pCR rate (68.18%), potentially inflating the observed efficacy. These findings reinforce CPS as a stratification tool in TNBC, and highlight the necessity of validating CPS thresholds in larger cohorts and incorporating diversified indexes, such as dynamic biomarkers, to optimize patient selection.

It is noteworthy that the other subgroup analyses failed to demonstrate statistically significant associations with pCR rates. For T stage, 79.54% (35/44) patients were at T2 stage, while 2 patients at T1 and 3 patients at T4. The uneven distribution of patients across subgroups may restrict the statistical power. Though the median age (48 years) of TREND trial is consistent to KEYNOTE-522, 93.2% (41/44) patients were younger than 65 years old. Immune responses in younger patients are stronger despite their aggressive disease. T cells with higher plasticity in younger patients provides a more responsive TME to immunotherapy. Younger patients were proved to benefit much from immunotherapy than the aged both in eTNBC and NSCLC.^24,25^ Hence, we would not overinterpret our results which showed a better pCR rate in patients under the age of 65. Indeed, the aged can also benefit from immunotherapy, which may be related to their higher accumulated TMB. Collectively, large-scale studies with broader and more evenly distributed populations are still needed to confirm these findings.

In addition to CPS, we performed DEG and GSEA analysis to explore the predictive indexes of response to neoadjuvant immunotherapy. We discovered that immune response-related pathways, including TNF signaling pathway, were enriched in pCR group before neoadjuvant. TNF signaling pathway plays a dual role in both promoting infiltration of lymphocytes and inducing immune evasion. *NECTIN4* could promote the lymphangiogenesis via CXCR4/CXCL12 axis, and antibody-drug conjugates (ADC) targeting *NECTIN4* have been utilized for the treatment of solid tumors.^26^ *GABRP* enhanced infiltration of macrophage through CXCL5/CCL20, facilitating tumor progression.^27^ Oncogenes highly expressed before therapy tended to remodel TME and sensitize tumor cells to immunotherapy. Conversely, elevated expression of genes involved in lipid metabolism was related to poor response of immunotherapy, demonstrating that the intervention in lipid metabolism may be the promising strategy for primary resistance to immunotherapy. Dynamic analysis suggested that the immune response enhanced at an early stage during neoadjuvant immunotherapy, demonstrating that an early evaluation of clinical and pathological changes might be a better choice.

Other investigations have reported that Tpex and CXCL13+ T cells are expanded in primary tumors of responsive patients, but not in those of unresponsive patients.^18,28^ Additionally, we found that CDKN1A+ CD8 T lymphocytes showed significant relevant with clinical response in this study. *CDKN1A*, also known as p21, is a gene that could control cell cycle.^29^ Deficiency of CDKN1A could inhibit exhaustion of Th1 cells and reduce cancer-related survival in colorectal cancer, which can be reversed by cyclin-dependent kinase (CDK) inhibitors such as palbociclib.^30^ Similarly, we found that CDKN1A + CD8 T lymphocytes exhibited low-level of exhaustion with high stemness and memory. These results suggested that neoadjuvant tislelizumab may promote effective antitumor response by enhanced CDKN1A+ CD8 T lymphocytes. Collectively, our data support the clinical potential of CDKN1A+ CD8 T lymphocyte as a predictive cell subset. Patients with enrichment of CDKN1A+ CD8 T lymphocytes was the specific population of TNBC that could benefit from neoadjuvant immunotherapy. And a combination of immunotherapy with CDK4/6 inhibitors could be explored in patients who were non-responsive to immunotherapy with a low level of CDKN1A+ CD8 T lymphocytes.

Tumor draining lymph nodes (tdLNs) were the location where dendritic cells primarily present antigens and T cells priming and proliferating.^31,32^ Therefore, tdLNs are the direct target of anti-PD-(L)1 and have been shown to play a key role in immunotherapy,^33–35^ although the response evaluation criteria of immunotherapy commonly used are still focused on the change of primary lesions and reported only tpCR and bpCR. The current prospective study found that the pCR rate of lymph nodes (ypN0) was extremely high (84.09%), since 37 of 44 patients in the EES were diagnosed as N1 or N2 stage before neoadjuvant therapy. Notably, the lymph nodes had higher TCR diversity in pCR patients, providing enriched TCR pool for effective immune response and disease control even after neoadjuvant therapy. However, despite effectively shrinking primary tumors, traditional neoadjuvant chemotherapy has not shown sufficient efficacy to warrant its use for treating metastatic lymph nodes. Our results illustrated that the addition of ICI to neoadjuvant chemotherapy might provide an effective option for reducing N stage in TNBC patients with metastases to lymph nodes, potentially avoiding resection of axillary lymph nodes, and preserving more tdLNs to promote long-term disease control. Notably, as the target organs of immunotherapy, lymph nodes also deserve consideration when evaluating the efficacy of neoadjuvant immunotherapies.

This study has several limitations. The single-arm and single-center design resulted in limited sample sizes and the lack of a control group, which precludes definitive conclusions about whether the observed efficacy is driven by tislelizumab, the chemotherapy backbone, or their synergistic effects. Consequently, we were unable to perform a direct comparison between our findings and historical data from trials such as KEYNOTE-522, as such comparisons are inherently speculative and potentially confounded by critical differences in chemotherapy regimens (platinum-free versus platinum-containing), baseline patient characteristics, and biomarker assessment methodologies. Additionally, samples applied for multi-omics analysis was restricted by tissue availability during trials, leading to selection bias. Furthermore, the follow-up duration was not sufficiently long to assess long-term outcomes. Large-scale randomized trials are urgently needed to determine the most suitable chemo-backbone for ICI and to identify the patient population who will receive the greatest benefit from immunotherapy. To address these limitations, randomized phase III trials stratified by biomarkers (*e.g*., PD-L1 CPS, and CDKN1A+ CD8 T lymphocytes) are awaited, comparing tislelizumab plus nab-P-EC against standard platinum-based regimens.

In conclusion, neoadjuvant tislelizumab combed with platinum-free and low-dose chemotherapy shows a favorable pCR rate in early TNBC, with an acceptable safety profile. Immune response was activated early in pCR patients, and enriched CDKN1A+ CD8 T lymphocytes after the NAT may be associated with the benefit from treatment. Future randomized controlled trials are warranted to confirm these findings.

## Materials and methods

### Ethics approval and consent to participate

This study involves human participants and was approved by Institutional Review Board of Liaoning Tumor Hospital (number: 20200622). Participants gave informed consent to participate in the study before taking part.

### Study design

TREND is a prospective single-arm, single-center, phase 2 study of tislelizumab and nab-P-EC with long-term follow-up in untreated TNBC patients with node-positive or at least T2 disease without distant metastasis clinical diagnosis. The study was approved by the institutional review boards at Liaoning Tumor Hospital (trial number: ChiCTR200035262) and was conducted in compliance with ethical standards and good clinical practice. Patients were recruited from the Liaoning Tumor Hospital from Nov 2020 to June 2023. All participants provided written informed consent in accordance with the Declaration of Helsinki v.2013. The Clinical Trials Office of Liaoning Tumor Hospital served as the coordinating center for the study. All information collected from study participants was kept confidential by assigning a random number to each patient, following institutional guidelines and policies.

### Study population

Enrolled patients had a documented diagnosis of TNBC, with eligibility based on 1) minimum tumor size of ≥2.0 cm regardless of lymph node status; or 2) lymph node positive regardless of tumor size; 3) without distant metastasis; and 4) no previous treatment. Populations were stratified by lymph node status, tumor size and PD-L1 status. All patients were female, 18 years of age or older, and had an Eastern Cooperative Oncology Group (ECOG) performance status of 0 or 1. Participants were excluded if they 1) experienced any distant metastases, 2) had prior therapy targeting immune checkpoint pathways, autoimmune disease, immunodeficiency, or immuno-suppressant use, or 3) active virus or bacterial infection. Participants received no financial or other compensation than the treatment for participating in the study.

### Treatments

All enrolled patients received a combination therapy of chemotherapy and tislelizumab for eight cycles (three weeks per cycle). Tislelizumab (200 mg) was administered intravenously (IV) at the first day of each cycle. During the first four cycles, patients received nab-P (100 mg/m^2^, IV) on the first day of each week, followed by a combination of epirubicin (75 mg/m², IV) and cyclophosphamide (600 mg/m², IV) on the first day of each three weeks for the last four cycles. Upon completion of the eight cycles of combined therapy, patients received surgery within 2 to 4 weeks (Fig. 1a). Surgery type was determined by the surgeon, including breast-conserving surgery or mastectomy for affected breasts, and sentinel lymph node biopsy or lymph node dissection for axillary lymph nodes.

### Study assessments

Pre-treatment biopsy specimens were assessed by histopathology to confirm diagnosis and to compare pre-treatment tumor morphology with any post-treatment residual tumor. Pathologic response was assessed in the post-treatment surgical specimens according to standard pathologic evaluation recommendations and reviewed by a dedicated pathologist to standardize reporting. pCR was defined as the absence of viable tumor in the post-treatment surgical specimens.

Tumors were assessed once every cycle using ultrasound imaging and every 2 cycles using magnetic resonance imaging following RECIST v.1.1, with confirmation by the principal investigator and radiology staff. ORR was defined as the percentage of patients with the best overall CR and PR, as per RECIST v1.1, requiring confirmation of CR or PR by repeat confirmation by imaging for at least four weeks after the initial assessment.

Adverse event (AE) seriousness, severity grade, and relationship to study treatment were assessed by physical examination and laboratory tests before every cycle, during follow-up visits, and upon indication by symptoms. Severity and grade were assessed by the treating physician, who determined whether AE was related to immunotherapy and/or chemotherapy, following definitions in the National Cancer Institute Common Terminology Criteria for Adverse Events v.5.0. All grades of AE were monitored and managed per protocol. Grade ≥ 2 irAEs were managed according to ASCO and CSCO guidelines. Patients with hepatitis or pneumonitis received corticosteroid therapy (prednisone 1 mg/kg/day), tapered gradually over 4 weeks upon clinical improvement. Immunotherapy was withheld until irAEs resolved to grade ≤ 1, while permanently discontinued for patients whose irAEs progressed to grade ≥ 4. Liver function tests and chest CT were performed twice per week until resolution. Neutropenia required granulocyte colony-stimulating factor (G-CSF) support.

### Study endpoints

The primary endpoint was pCR, defined as the pathological stage of ypT0/Tis ypN0 during the final surgery. The secondary endpoint was the ORR according to RECIST v1.1 and safety. The exploratory endpoints included potential correlations between efficacy and PD-L1 status, immune status of TME, and/or genetic profile.

### Analysis sets

The SS included all enrolled patients who received at least one treatment cycle. The EES included all patients who received neoadjuvant tislelizumab plus chemotherapy, underwent documented surgery, and had available pathological efficacy without major protocol deviation. The primary analysis was performed in the EES, while patient disposition and all safety analyses were performed in the SS.

### Sample collection

Tumor samples were collected from biopsies obtained from at least one tumor site before administration of the first therapy dose, after cycle one and at the time of surgery.

CPS and TPS and of PD-L1 protein expression were analyzed in pre-neoadjuvant biopsies by the Department of Pathology in the Liaoning Tumor Hospital using a PD-L1 IHC 22C3 pharmDx assay (Agilent). Blood samples were collected at screening, every week upon treatment, and at the end of treatment or disease progression.

To address the objectives of identifying predictive biomarkers and immunologic mechanisms, complementary multi-omics approaches were prioritized. ScRNA-seq was applied to profile the heterogeneity of TME and identify immune subsets related to response. TCR-seq enabled tracking of clonal T-cell dynamics, linking repertoire expansion to clinical outcomes. CyTOF was utilized for its high-dimensional protein-level profiling capacity to profile T-cell functional states. Bulk RNA-seq enabled pathway enrichment analysis across the entire process of neoadjuvant therapy respectively, while WES was performed to describe the tumor mutation burden. Subset information of the patients and samples for different sequencing analysis were listed in Supplementary Table S3, while clinical and pathological characteristics at baseline for above subsets were showed in Supplementary Table S4.

### Study termination

The pCR for this patient population was 41% with platinum-free nab-P-EC-based neoadjuvant chemotherapy, as reported in IMpassion-031.^5^ This benchmark was selected to evaluate whether tislelizumab combined with a platinum-free backbone could surpass chemotherapy-alone efficacy, independent of platinum’s contribution. Thus, we estimated that pCR would improve to 56% with the ICB combined treatment. Patients were excluded from the statistical analysis if they did not meet the above eligibility criteria. A sample size of 65 patients was estimated using a one-arm design with a null hypothesis of pCR≤ 0.41 versus its one-sided alternative. A group sequential design based on the two classification endpoints of a single arm trial was adopted, and the trial was designed to accommodate an interim analysis after enrolling 48 patients. A finding of futility was based on a Bayesian optimal phase 2 design with one interim analysis. If 20 or fewer responses were observed, the trial would stop earlier. If the number of patients who achieved pCR was greater than or equal to 30, accrual could be terminated early after reaching the primary endpoint. This design yielded a one-sided type 1 error of 0.05 and a power of 80.0% when the true response rate was 56%. It should be noted that the TREND trial was terminated early due to meeting the primary endpoint.

### Statistical analysis

For measurement data, we present mean, standard deviation, median, maximum, and minimum values. For enumeration and ranked data, we provide frequency (constituent ratio), rates, and confidence intervals. The SPSS 23.0 Statistical Analysis Software package was used for all statistical analyses, with significance determined by two-sided T-tests at a significance level of *P* ≤ 0.05 and a 95% confidence interval. The measurement data for each patient collected at each visit are presented as means ± SD or as medians (minimum, maximum). Paired t-tests were used to compare pre- and post-treatment parameters. Subject measurement data acquired in each visit are shown as frequency (constituent ratio). Chi-square tests, Fisher’s precision probability tests, or non-parametric tests were used to assess changes between pre- and post-treatment measurements.

### RNA extraction, library preparation and sequencing

Total RNAs were extracted from tissue or cells samples using TRIzol (Invitrogen™, Cat. No. 15596018). About 0.5 ~ 1 μg total RNA of each sample were used for TCR sequencing library preparation using KC-DigitalTM Stranded TCR-seq Library Prep Kit for Illumina® 150 (Seqhealth Technology Co., Ltd., Wuhan, China, Cat. No. DT0813-02) following the manufacturer’s instruction. The kit eliminates duplication bias in polymerase chain reaction and sequencing steps, by using unique molecular identifier (UMI) of 8 random bases to label the pre-amplified cDNA molecules. The library products corresponding to 250-500 bp were enriched, quantified and finally sequenced on NovaSeq (Illumina®).

### RNA-Seq data analysis

Raw sequencing data was first filtered by fastp, low-quality reads and the reads contaminated with adaptor sequences were discarded. Clean Reads were further treated with in-house scripts to eliminate duplication bias introduced in library preparation and sequencing. UMI was utilized to eliminate errors and biases introduced in sequencing process. In brief, clean reads were first clustered according to the UMI sequences, in which reads with the same UMI sequence were grouped into the same cluster. Reads in the same cluster were compared to each other by pairwise alignment, and then reads with sequence identity over 95% were extracted to a new sub-cluster. After all sub-clusters were generated, multiple sequence alignment was performed to get one consensus sequence for each sub-cluster.

The de-duplicated consensus sequences were used for TCR-seq analysis. They were mapped to the international ImMunoGeneTics database to obtain V, D and J fragment, rearrangement and CDR3 sequences. Further data statistics and analysis were performed using VDJtools and immunarch software. Detailed software versions, databases, and parameters are provided in Supplementary Methods.

### Time course RNASeq data analysis

We employed a time-series analysis approach to examine multi-timepoint RNA sequencing data. Upon implementing rigorous quality control measures and normalization of the data to account for technical variability, we performed differential expression analysis to identify genes with significant changes in expression over time. k-means clustering is performed, which is an unsupervised machine learning algorithm that partitions data points into k predefined groups by iteratively minimizing the distance between points and cluster centroids, ensuring high intra-cluster similarity and inter-cluster distinction. By employing a statistical framework that accounts for time-dependent structure in the data, we were able to discern patterns of gene expression that evolved during the course of the experiment. Subsequent to differential expression analysis, we conducted clustering of the time-course data based on spline fitting and the fuzzy c-means clustering algorithm. Fuzzy c-means algorithm is a soft clustering method that assigns each data point probabilistic membership scores across multiple clusters, thereby capturing overlapping expression patterns inherent in heterogeneous biological systems. This enabled the grouping of genes witAedimilatimorression profiles over time, which may suggest a shared regulatory mechanism or involvement in a common biological pathway. Finally, to glean insights into the biological significance of our findings, we performed Gene Ontology (GO) and the Kyoto Encyclopedia of Genes and Genomes (KEGG) enrichment analysis using the clusterProfiler R package to detect comprehensive biological functions and pathways among the genes within each cluster. Detailed software versions, and R package are provided in Supplementary Methods.

### Single-cell RNA-seq data processing and annotation

The Cell Ranger toolkit provided by 10x Genomics was applied to align reads to human reference genome (GRCh38) and generate the UMI matrix. After UMI matrix generation, doublet score of each cell was predicted by Scrublet,^36^ and 0.3 was used as a cut-off to filter out doublets. The R-based toolkit, Seurat,^37^ was used for downstream analysis of scRNA-seq data. We further kept high-quality cells with thresholds of 1,000 – 25,000 UMIs, 500-5000 genes and less than 10% mitochondrial gene counts.

After quality control, the raw count matrix was normalized using *NormalizeData()* with LogNormalize as normalization method and then scaled by 10,000 and logarithmically transformed. A total of 2000 high variable genes were selected by *FindVariableFeatures()*. The percent of mitochondrial genes and UMIs of each cell were regressed out during scaling. The top 50 PCs were calculated, and then batch effect was removed with Harmony^38^ across different patients. Nearest neighborhood graphs were build using *FindNeighbors()*, and the community algorithm was applied for clustering using the Louvain function with resolution = 1. For visualization, the dimensionality was further reduced by t-distributed Stochastic Neighbor Embedding (t-SNE). Detailed software versions, and R package are provided in Supplementary Methods.

### Single-cell TCR-seq data analysis

The TCR sequence data from 10X Genomics were processed using Cell Ranger toolkit with human VDJ reference genome (GRCh38). For untreated Pt4, due to the total reads and mapping rates of metastatic lymph notes -1 were not pass the standard, it was removed in the downstream analysis. For other samples, the output file filtererd_contig_annotations.csv, which containing TCR α- and β-chain CD3 nucleotide sequences, was highly confident, full length, with a valid cell barcode and an unambiguous chain type assignment were retained. If a cell had two or more qualified chains of the same type, only that chain with the highest UMI count was qualified and kept. The contig annotation data of each sample was merged using the combineTCR() function of scRepertoire.^39^ For each patient, the cells with an identical protein sequence of α- and β- chain were marked as same clonetype. Only the cells with both scTCR-seq and scRNA-seq data were applied to further clonetype-related analysis. Detailed software versions, and parameters are provided in Supplementary Methods.

### CyTOF analysis of immune cells

The samples were collected and Live/Dead stained with 2 μM cisplatin (Fluidigm) for 2 minutes before quenching with CSB (Fluidigm). A Fix-I buffer (Fluidigm) was then used to fix cells for 15 minutes at room temperature, followed by washing three times with 1x PBS. The samples were stained with Cell-ID™ 20-Plex Pd Barcoding Kit (Fluidigm) to minimize internal cross reaction between samples. MaxPar × 8 Polymer Kits (Fluidigm) were used to conjugate with purified antibodies according to the manual. All metal-conjugated antibodies were titrated for optimal concentrations before use. For the surface protein staining, cells were counted, diluted into 1× 106 cell/ml in PBS and cultured with antibodies cock-tail in a total 50 μL CSB for 30 minutes at RT. After that, cells were washed, underwent permeabilization with 80% methanol for 15 minutes at °C and stained with an intracellular antibody cocktail for 30 minutes. After triple washes in CSB, cells were triple washed in CSB and incubated with 0.125 μM iridium intercalator in fix and perm buffer (Fluidigm) at 4 °C overnight.

After cultured with intercalator, cells were washed with ice cold PBS and deionized water three times separately. Prior to acquisition, samples were resuspended in deionized water containing 10% EQ 4 Element Beads (Fluidigm) and cell concentrations were adjusted to 1×106 cell/ml. Data acquisition was performed on a Helios mass cytometer (Fluidigm). The original FCS data were normalized and. fcs files for every sample were collected. All. fcs files were uploaded into Cytobank, data cleaning and populations of single living cells were exported as .fcs files for further analysis.

### WES

Tumor tissue samples were processed to simultaneously isolate genomic DNA and total RNA using the QIAamp AllPrep DNA/RNA mini-Kit (Qiagen, catalog no. 80204) following the prescribed protocol, which encompassed the separation, purification, and elution of DNA and RNA through column-based methods. For peripheral blood samples, genomic DNA extraction was performed with the QIAamp DNA mini-Kit (Qiagen, catalog no. 51304) according to the manufacturer’s guidelines. The concentration of the extracted DNA was determined using the Qubit dsDNA BR Assay Kit (Thermo Fisher Scientific, catalog no. Q32850), while the integrity of the DNA was assessed via agarose gelelectrophoresis. Whole-exome libraries were constructed using a MGIEasy Exome Universal Library Prep Set (MGI, catalog no. 1000009657) as per the manufacturer’s protocol. This involved fragmenting the DNA, ligating adapters, conducting probe hybridization, and performing PCR amplification. The quality of the libraries was evaluated using a Qubit dsDNA HS Assay Kit (Thermo Fisher Scientific, catalog no. Q32851) and Agilent DNA 1000 Kit (Agilent, catalog no. 5067-1504). Subsequently, the libraries were sequenced on the DNBSEQ T1 platform (MGI) with 100 bp paired-end reads. The sequencing coverage achieved a mean depth of ×435 for tumor samples and ×212 for peripheral blood.

## Supplementary information

Supplemental material

study protocol

## Data Availability

The raw sequencing data reported in this paper will be deposited in the Genome Sequence Archive (GSA) at the National Genomics Data Center, China National Center for Bioinformation/Beijing Institute of Genomics, Chinese Academy of Sciences and be publicly accessible at https://ngdc.cncb.ac.cn/gsa. In accordance with the Regulation of the People’s Republic of China on the Administration of Human Genetic Resources, access to the Human Genetic Resource Data requires an application to the Data Access Committee for research purposes. Users must submit a data access request, which will be approved upon meeting the necessary criteria. The data will then be made available upon reasonable request, subject to the terms of consent and data use limitations for the subjects. For further inquiries, please contact the corresponding author.
