# Supplementary material for: Efficacy, safety and exploratory analysis of neoadjuvant tislelizumab (a PD-1 inhibitor) plus nab-paclitaxel followed by epirubicin/cyclophosphamide for triple-negative breast cancer: a phase 2 TREND trial
Source: Signal Transduct Target Ther. 2025 May 26;10:169. doi: 10.1038/s41392-025-02254-3 (PMC12104340; doi:10.1038/s41392-025-02254-3)
Supplement: Supplementary file 1 — Supplemental material [file 41392_2025_2254_MOESM1_ESM.docx]

Supplementary Materials for

Efficacy, safety and exploratory analysis of neoadjuvant tislelizumab plus nab-paclitaxel followed by epirubicin/cyclophosphamide for TNBC: a phase 2 TREND trial

Qiang Zhang, Mozhi Wang, Yumeng Li, Hengjun Zhang, Yusong Wang, Xiuyun Chen, Litong Yao, Mingke Cui, Haoran Dong, Xiang Li, Jian Liu, Bo Zhu, and Yingying Xu

Correspondence to: xuyingying@cmu.edu.cn; Bo.zhu@tmmu.edu.cn

**This PDF file includes:**

Materials and Methods

Supplementary Text

Figures. S1 to S4

Tables S1 to S4

Materials and Methods

Methods

**Detailed Software Versions and Parameters.**

| **Section** | **Tool/Software** | **Version** | **Description** |
| --- | --- | --- | --- |
| RNA extraction, library preparation and sequencing | NovaSeq | - | Illumina® |
| RNA-Seq data analysis | fastp | 0.23.1 | Low-quality reads and adaptor contamination filtering |
|  | kcUID | - | In-house scripts for duplication bias elimination |
|  | MiXCR | 3.0.3 | TCR-seq analysis (mapping to ImMunoGeneTics database) |
|  | VDJtools and immunarch | - | Data statistics and analysis |
| Time Course RNASeq Data Analysis | R package moanin and Mfuzz | - | Clustering of time-course data (spline fitting and fuzzy c-means clustering) |
|  | clusterProfiler R package | - | Gene Ontology (GO) and KEGG enrichment analysis |
| Single-cell RNA-seq data processing and annotation | Cell Ranger toolkit | 6.1.2 | Alignment to human reference genome (GRCh38) and UMI matrix generation |
|  | Scrublet | - | Doublet score prediction |
|  | Seurat | 4.3.0 | Downstream analysis of scRNA-seq data |
|  | Harmony | - | Batch effect removal across different patients |
| Single-cell TCR-seq data analysis | Cell Ranger toolkit | 6.1.2 | Processing of TCR sequence data (human VDJ reference genome GRCh38) |
|  | scRepertoire | 1.5.2 | Merging contig annotation data and clonetype analysis |
| Whole exome sequencing | BWA | 0.7.12 | Align WES data to the hg38 reference genome |
|  | Picard | 1.84 | Remove duplicate reads |
|  | GATK | 4.1 | Local realignment and base quality score recalibration |
|  | SomaticSniper | 1.0.5.1 | Detect single nucleotide variants, small insertions and deletions |
|  | MutTect2 | 2.7.0 |  |
|  | MuSE | 1.0 |  |
|  | Strelka | 2.9.9 |  |
|  | Svaba | 0.2.1 |  |
|  | deconstructSigs | 1.8.0 | Determine mutational signatures |
|  | FACETS | 0.16.0 | Copy number variants, tumor purity and ploidy |
|  | GISTIC | 2.0.23 | Identify significantly amplified or deleted regions of copy number variants |

Materials

**Antibodies and reagents for cyTOF**

| **Reagent or Resource** | **Source** | **Identifier** | **Clone** | **Label** |
| --- | --- | --- | --- | --- |
| **Antibodies** | | |  |  |
| CD45 | fluidigm | 304045 | BC8 | X89Y |
| CD19 | fluidigm | 302202 | HIB19 | X141Pr |
| CD138 | fluidigm | H04711TBD-50T | DL-101 | X176Lu |
| CD3 | fluidigm | 14-0031-82 | UCHT1 | X170Yb |
| CD56 | fluidigm | 318302 | NCAM16.2 | X159Tb |
| CD4 | fluidigm | 14-0049-80 | OKT4 | X116Cd |
| CD8 | fluidigm | MHCD0800 | UCHT4 | X168Er |
| CD25 | fluidigm | 14-0259-82 | M-A251 | X149Sm |
| CD127 | fluidigm | H03612094-25T | A019D5 | X143Nd |
| γδTCR | fluidigm | H40762098-25T | REA591 | X152Gd |
| CD45RA | fluidigm | H21411112-25T | HI100 | X146Nd |
| CD62L | fluidigm | H24111107-25T | DREG56 | X155Gd |
| CD45RO | fluidigm | H21612105-25T | UCHL1 | X166Er |
| PD-1 | fluidigm | H15811111-25T | EH12.2H7 | X151Eu |
| TIM-3 | fluidigm | H20311138-25T | F38-2E2 | X142Ce |
| LAG-3 | fluidigm | 125202 | 11C3C65 | X175Lu |
| CD69 | fluidigm | H25021TBD-50T | FN50 | X153Eu |
| Ki67 | fluidigm | AB279657 | B56 | X172Yb |
| CD68 | fluidigm | 333802 | BL13756 | X173Yb |
| HLA-DR | fluidigm | H31111111-25T | L243 | X156Gd |
| CD86 | fluidigm | 14-0862-82 | IT2.2 | X147Sm |
| CD206 | fluidigm | H11321TBD-50T | 15-2 | X161Dy |
| CD14 | fluidigm | H04842127-25T | UCHM1 | X160Dy |
| CD11c | fluidigm | 301601 | BU15 | X162Dy |
| CD123 | fluidigm | 306010 | 6H6 | X171Yb |
| CD15 | fluidigm | H05612TBD-50T | W6D3 | X148Sm |
| CD66b | fluidigm | 392902 | 6/40C | X174Yb |
| Vimentin | fluidigm | N/A | N/A | X154Gd |
| CD105 | fluidigm | N/A | N/A | X163Dy |
| CD31 | fluidigm | 102425 | N/A | X144Nd |
| PDPN | fluidigm | AB10288 | N/A | X165Ho |
| CD21 | fluidigm | 354902 | N/A | X145Nd |
| CTLA4 | fluidigm | H05812099-25T | L3D10 | X158Gd |
| OX40 | fluidigm | H04311109-25T | ACT35 | X150Sm |
| 4-1BB | fluidigm | N/A | N/A | X209Bi |
| CXCR5 | fluidigm | H09131125-25T | J252D4 | X164Dy |
| SLAMF6 | fluidigm | AB246707 | N/A | X167Er |
| **Chemicals, peptides and recombinant proteins** | | |  |  |
| 191Ir | fluidigm | 201192A |  |  |
| 193Ir | fluidigm | 201192A |  |  |
| Cell-ID Cisplatin | fluidigm | 201064 |  |  |

Supplementary Text

Detailed methodologies for figures

**Figure 1. Trial design.**

(a) Study design for the TREND trial, (b) Flowchart for patient enrollment.

**Figure 2. Efficacy of TREND trial in EES.**

(a) pCR in primary tumors (ypT0/is) and axillary lymph nodes (ypN0) after neoadjuvant treatment, (b) Relationship between tpCR rate and CPS (left, CPS threshold =10; right, CPS threshold = 20), (c) Representative images of H&E staining in one pCR patient (no.12) and one non-pCR patient (no.10) before (left) and after (right) neoadjuvant treatment, (d) Waterfall plots of the best clinical response based on RECIST V1.1 definitions of changes in tumor size from pre-neoadjuvant to post-neoadjuvant stage in EES, (e) Magnetic resonance imaging of representative clinical responses in two pCR patients (no.24, no.47) and two non-pCR patients (no.10, no.13) before and after neoadjuvant treatment. pCR, pathological complete response; EES, efficacy evaluable set; RECIST, Response Evaluation Criteria in Solid Tumors; CR, complete response; PR, partial response; SD, stable disease; PD, progressive disease; CPS, combined positive score; H&E, hematoxylin and eosin. Data cutoff: June 30, 2023.

**Figure 3. Gene expression profile before neoadjuvant therapy and predictive index of response.**

(a) The volcano plot of transcriptomic characteristics between pCR and non-pCR patients at baseline (pre-neoadjuvant), (b–f) Gene set enrichment analysis of the Hallmark TNF, T cell receptor and JAK-STAT signaling pathway, PD-L1/PD-1 checkpoint pathway in cancers and cytokine-cytokine receptor interaction, (g) Box plot of the predictive score between pCR and non-pCR patients in training cohort. Training cohort includes 7 samples from TREND and 29 samples from the TNBC cohort of ISPY-2 that received pembrolizumab-combined chemotherapy. 23 for pCR and 13 for non-pCR. (h) The ROC curve of the predictive model in training cohort, (i) Box plot of the predictive score between pCR and non-pCR patients in validation cohort. Validation cohort includes 21 samples from the TNBC cohort of ISPY-2 that received durvalumab-combined chemotherapy. 9 for pCR and 12 for non-pCR. (j) The ROC curve of the predictive model in training cohort. pCR, pathological complete response; TNF, tumor necrosis factorJ; NES, normalized enrichment score; JAK, Janus Kinase; STAT, signal transducer and activator of transcription; PD-L1, programmed cell death 1 ligand 1; PD-1, programmed cell death-1; AUC, area under the curve; ROC curve, receiver operating characteristic curve.

**Figure 4. Dynamic change of TME during neoadjuvant tislelizumab and chemotherapy**

(a) The volcano plot of transcriptomic characteristics between before and after neoadjuvant therapy in pCR patients, (b) The volcano plot of transcriptomic characteristics between before and after neoadjuvant therapy in non-pCR patients, (c) the relationship of pCR rate and dynamic change of ki67 from baseline,△ki67= ki67 at baseline to C1D1(Day1 of Cycle1, (d) ssGSEA analysis of RNA-seq data from tumor samples at baseline (C0), (e) heatmap of genes in cluster 5 enriched according to k-means clustering based on spline fitting, (f-g) GO and KEGG enrichment analysis of genes in cluster 5 enriched according to k-means clustering based on spline fitting, and cluster 1 and 3 in pCR group and cluster 2 and 7 in non-pCR group based on the clustering of fuzzy c-means algorithm. pCR, pathological complete response; NAT, neoadjuvant therapy; C0D1, Day1 of Cycle0 (C0, baseline); C1D1, Day1 of Cycle1; GSVA, gene set variation analysis; ssGSEA, single sample gene set enrichment analysis; GO, gene ontology; BP, biological process; CC, cellular component; MF, molecular function; KEGG, Kyoto Encyclopedia of Genes and Genomes.

**Figure 5. Transcriptional and clonal diversity of CD8 T lymphocytes between pCR and non-pCR patients**

(a) tSNE of cells from total samples colored by cell type based on scRNA-seq, (b) Expressions of selected canonical marker genes in each major cell population based on scRNA-seq, (c) tSNE of CD8 T lymphocytes from total samples colored by cell type based on scRNA-seq, (d) Expressions of selected canonical marker genes in each major CD8 T lymphocytes populations based on scRNA-seq, (e)Abundance of major CD8 T lymphocytes populations in each tissue sample based on scRNA-seq, (f) Heatmap and abundance of major CD8 T lymphocytes populations in all primary tumors based on cyTOF data, (g) Validation of CDKN1Ahi CD8 T lymphocytes score in pCR and non-pCR patients based on RNA-seq data. (h) Volcano plot showing differentially expressed genes between CDKN1A+ CD8 T lymphocytes and CDKN1A- CD8 T lymphocytes, (i) External validation of CDKN1Ahi CD8 T lymphocytes in pCR and non-pCR patients based on cohort 1 from BioKey, (j) External validation of CDKN1Ahi CD8 T lymphocytes in pCR and non-pCR patients based on GSE246613, (k) Venn plot showing numbers of the shared TCR clonetypes of CD8 T lymphocyte between primary lesions and lymph nodes. Hypergeometric test, (l) Shannon Index of TCR in tumor, peripheral blood and lymph nodes between pCR and non-pCR patients, (m) TCR clonality in tumor, peripheral blood and lymph nodes between pCR and non-pCR patients, (n) Gini Simpson index of TCR in tumor, peripheral blood and lymph nodes between pCR and non-pCR patients. tSNE, t-Distributed Stochastic Neighbor Embedding; pCR, pathological complete response. E, T cell expansion. NE, no T cell expansion.

Figure. S1.


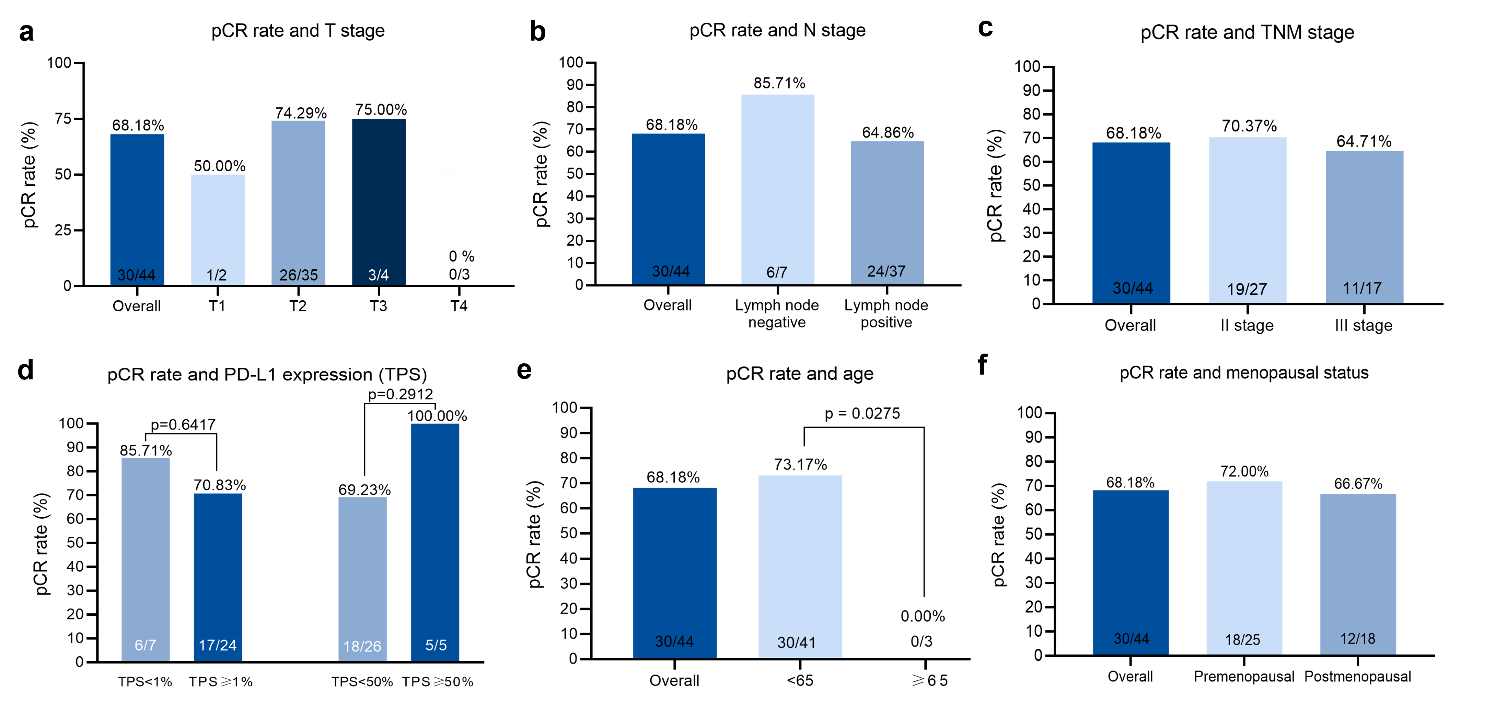


Subgroup analysis of pCR rate in EES. (a) Relationship between tpCR rate and T stage, (b) Relationship between tpCR rate and N stage, (c) Relationship between tpCR rate and TNM stage according to AJCC (eighth edition), (d) Relationship between tpCR rate and TPS (left, TPS threshold =1; right, TPS threshold = 50), (e) Relationship between tpCR rate and age, (f) Relationship between tpCR rate and menopausal status. pCR, pathological complete response; EES, efficacy evaluable set; tpCR, total pathological complete response; AJCC, American Joint Committee on Cancer; CPS, combined positive score; TPS, tumor proportion score. PD-L1 status was evaluated by CPS and TPS using PD-L1 IHC 22C3 PharmDx. Data cutoff: June 30, 2023

Figure. S2.


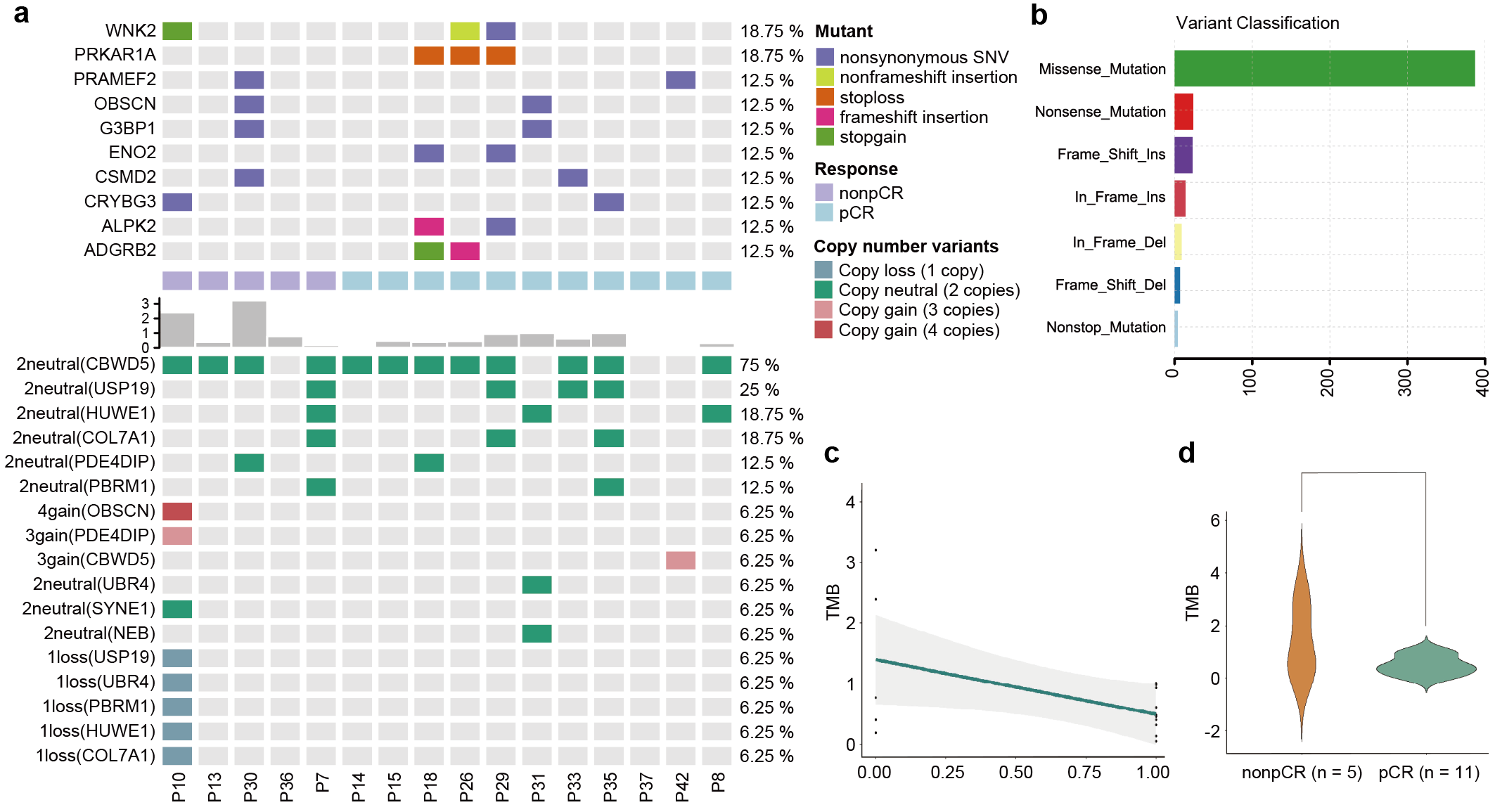


Mutational analysis before neoadjuvant tislelizumab combined with chemotherapy. (a) Genomic landscape before the neoadjuvant therapy. Columns represent individuals. Rows represent specific genes, (b) Waterfall plot of the variant classifications in pCR (n=11) and non-pCR patients (n=5), (c) Correlation of the tumor mutational burden between pCR (n=11) and non-pCR patients (n=5), (d) Violin plot of the tumor mutational burden in pCR (n=11) and non-pCR patients (n=5). pCR, pathological complete response.

Figure. S3.
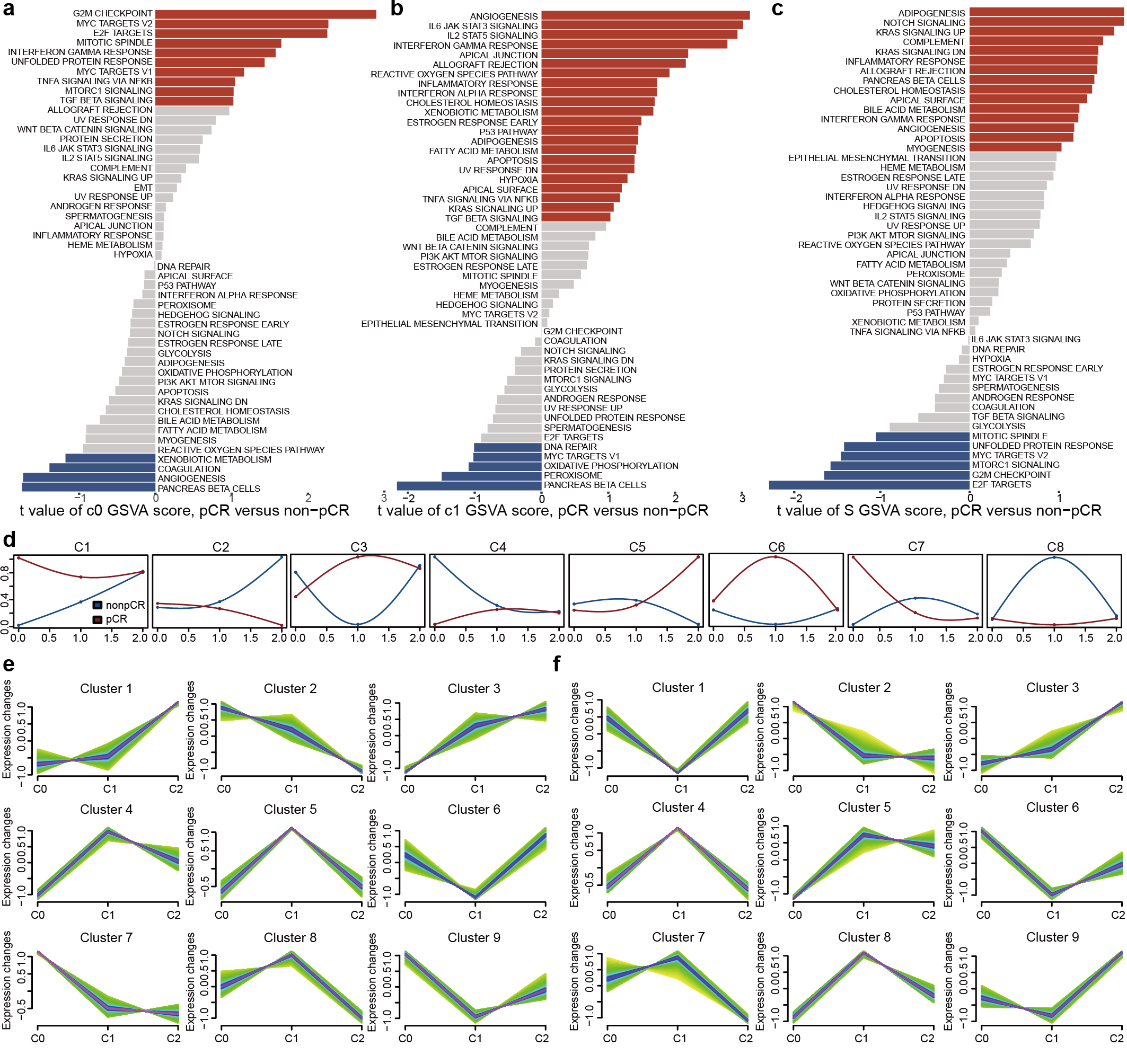


DEGs and pathways of dynamic change during NAT. (a-c) GSVA analyasis of RNA-seq data from pCR versus non-pCR samples at baseline (C0), C1 and S according to HALLMARKS pathway in Molecular Signatures database, (d) k-means clustering based on spline fitting and enrichment of 8 clusters of genes with similar characteristics, (e) the clustering of genes with similar features in pCR patients based on fuzzy c-means algorithm, (f) the clustering of genes with similar features in non-pCR patients based on fuzzy c-means algorithm. pCR, pathological complete response; GSVA, gene set variation analysis.

Figure. S4.


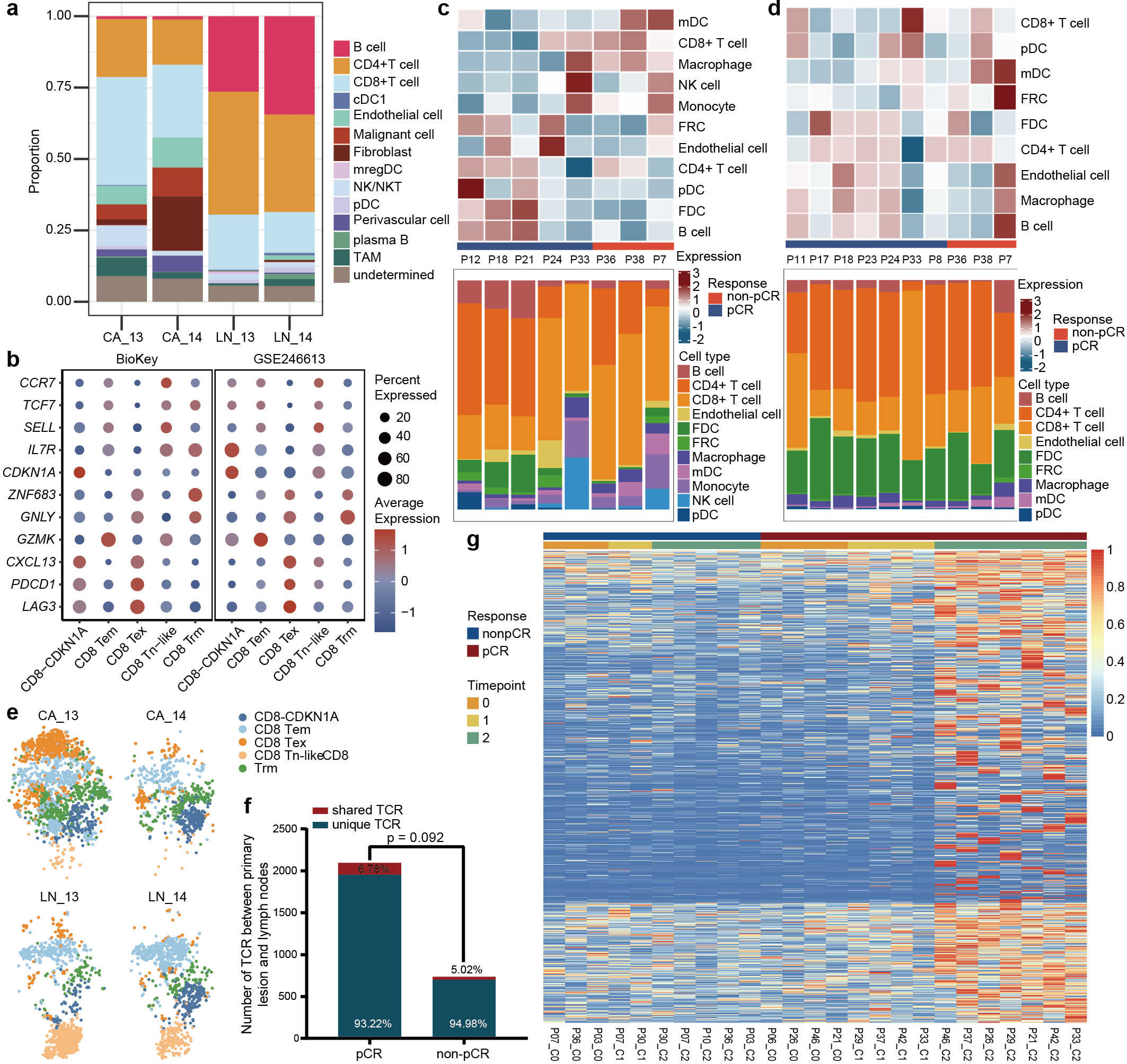


(a) Abundance of major cells populations in each tissue sample based on scRNA-seq, (b) Expressions of selected canonical marker genes in each major CD8 T lymphocytes populations based on scRNA-seq from cohort 1 of BioKey and GSE246613, (c) Heatmap and abundance of major cells populations in all primary tumors based on cyTOF data, (d) Heatmap and abundance of major cells populations in all lymph nodes based on cyTOF data, (e) tSNE of CD8 T lymphocytes from each sample colored by cell type based on scRNA-seq, (f) Statistical column plot showing relationship of pCR and numbers of the shared TCR clonetypes of CD8 T lymphocyte in primary tumor. Hypergeometric test, (g) Differences of the shared TCR clonetypes of CD8 T lymphocyte among primary tumor, lymph nodes and peripheral bloods. CA, primary tumor; LN, lymph nodes; pCR, pathological complete response; tSNE, t-Distributed Stochastic Neighbor Embedding; TCR, T cell receptor.

Table S1. Clinical characteristics at baseline (SS and EES population)

|  | **SS (*n* = 53)** | **EES (*n* = 44)** |
| --- | --- | --- |
| **Median age (min-max), years** | 48.51 (25 - 69) | 48.59 (25 - 69) |
| **Females, n (%)** | 53 (100.00) | 44 (100.00) |
| **Menopausal status, n (%)** |  |  |
| Premenopausal | 28 (52.83%) | 25 (56.82%) |
| Perimenopausal | 2 (3.77%) | 1 (2.27%) |
| Postmenopausal | 23 (43.40%) | 18 (40.91%) |
| **Median Ki67 (min-max)** | 55.85 (10 - 90) | 57.50 (20 - 90) |
| **T stage, n (%)** |  |  |
| T1 | 3 (5.66%) | 2 (4.55%) |
| T2 | 41 (77.36%) | 35 (79.55%) |
| T3 | 4 (7.55%) | 4 (9.09%) |
| T4 | 5 (9.43%) | 3 (6.82%) |
| **N stage, n (%)** |  |  |
| N0 | 8 (15.09%) | 7 (15.91%) |
| N1 | 31 (58.49%) | 25 (56.82%) |
| N2 | 14 (26.42%) | 12 (27.27%) |
| **AJCC stage, n (%)** |  |  |
| IIA | 10 (18.87%) | 8 (18.18%) |
| IIB | 24 (45.28%) | 19 (43.18%) |
| IIIA | 14 (26.42%) | 14 (31.82%) |
| IIIB | 5 (9.43%) | 3 (6.82%) |
| **CPS, n (%)** |  |  |
| < 1 | 2 (3.77%) | 2 (4.55%) |
| ≥ 1, < 10 | 13 (24.53%) | 12 (27.27%) |
| ≥ 10, ≤ 100 | 31 (58.49%) | 26 (59.09%) |
| Unknown | 7 (13.21%) | 4 (9.09%) |
| **TPS, n (%)** |  |  |
| < 1% | 7 (13.21%) | 7 (15.91%) |
| ≥ 1%, < 50% | 22 (41.51%) | 19 (43.18%) |
| ≥ 50% | 6 (11.32%) | 5 (11.36%) |
| Unknown | 18 (33.96%) | 13 (29.55%) |

Data cutoff: June 30, 2023. SS, safety analysis set; EES, efficacy evaluable set; AJCC, American Joint Committee on Cancer; CPS, combined positive score; TPS, tumor proportion score. PD-L1 status was evaluated by CPS and TPS using PD-L1 IHC 22C3 PharmDx.

Table S2. Disease response per RECIST v1.1 (EES population)

|  | **EES (n = 44)** |
| --- | --- |
| **Best overall response, *n* (%)** | |
| CR | 14 (31.82) |
| PR | 27 (61.36) |
| SD | 2 (4.55) |
| PD | 0 (0.00) |
| Could not be determined | 1 (2.27) |
| **ORR, % (95% CI)^a^** | 93.18, 80.29 – 98.22 |
| **DCR, % (95% CI)^b^** | 97.73, 86.49 – 99.88 |

Data cutoff: June 30, 2023. RECIST, Response Evaluation Criteria in Solid Tumors; EES, efficacy evaluable set; CR, complete response; PR, partial response; SD, stable disease; PD, progressive disease; ORR, objective response rate; CI, confidence interval; DCR, disease control rate. 95% CI was calculated by Wilson method.

^a^ ORR = CR + PR

^b^ DCR = CR + PR + SD

Table S3. Clinical and pathological information of the patient and sample in different analysis.

| **Analysis** | **Patient** | **Sample** | **Time** | **Pathological Response** |
| --- | --- | --- | --- | --- |
| **scRNA**-**seq and paired scTCR-seq** |  |  |  |  |
|  | Patient 13 | Primary lesion and two axillary lymph nodes | Surgery | ypT1 ypN0 |
|  | Patient 14 | Primary lesion and an axillary lymph node | Surgery | ypT0 ypN0 |
| **RNA-seq** |  |  |  |  |
|  | Patient 3 | Primary lesion | Baseline, surgery | ypT1 ypN0 |
|  | Patient 6 | Primary lesion | Baseline | ypT0 ypN0 |
|  | Patient 7 | Primary lesion | Baseline, one cycle, surgery | ypT1 ypN1 |
|  | Patient 10 | Primary lesion | Surgery | ypT1 ypN0 |
|  | Patient 21 | Primary lesion | Baseline, surgery | ypT0 ypN0 |
|  | Patient 26 | Primary lesion | Baseline, surgery | ypT0 ypN0 |
|  | Patient 28 | Primary lesion | One cycle, surgery | ypT0 ypN0 |
|  | Patient 32 | Primary lesion | One cycle, surgery | ypT0 ypN0 |
|  | Patient 35 | Primary lesion | Baseline, surgery | ypT1 ypN0 |
|  | Patient 40 | Primary lesion | One cycle, surgery | ypT0 ypN0 |
|  | Patient 43 | Primary lesion | Baseline, surgery | ypT0 ypN0 |
| **CyTOF** |  |  |  |  |
|  | Patient 7 | Primary lesion and axillary lymph node | Surgery | ypT1 ypN1 |
|  | Patient 12 | Primary lesion | Surgery | ypT0 ypN0 |
|  | Patient 18 | Primary lesion and axillary lymph node | Surgery | ypT0 ypN0 |
|  | Patient 21 | Primary lesion | Surgery | ypT0 ypN0 |
|  | Patient 24 | Primary lesion and axillary lymph node | Surgery | ypT0 ypN0 |
|  | Patient 32 | lymph node | Surgery | ypT0 ypN0 |
|  | Patient 35 | Primary lesion and axillary lymph node | Surgery | ypT1 ypN0 |
|  | Patient 37 | Primary lesion and axillary lymph node | Surgery | ypT0 ypN1 |
|  | Patient 8 | Axillary lymph node | Surgery | ypT0 ypN0 |
|  | Patient 11 | Axillary lymph node | Surgery | ypT0 ypN0 |
|  | Patient 17 | Axillary lymph node | Surgery | ypT0 ypN0 |
|  | Patient 23 | Axillary lymph node | Surgery | ypT0 ypN0 |
| **WES** |  |  |  |  |
|  | Patient 13 | Primary lesion and paired normal tissues | Surgery | ypT1 ypN0 |
|  | Patient 32 | Primary lesion and paired normal tissues | Surgery | ypT0 ypN0 |
|  | Patient 10 | Primary lesion and paired normal tissues | Surgery | ypT1 ypN0 |
|  | Patient 14 | Primary lesion and paired normal tissues | Surgery | ypT0 ypN0 |
|  | Patient 18 | Primary lesion and paired normal tissues | Surgery | ypT0 ypN0 |
|  | Patient 7 | Primary lesion and paired normal tissues | Surgery | ypT1 ypN1 |
|  | Patient 8 | Primary lesion and paired normal tissues | Surgery | ypT0 ypN0 |
|  | Patient 15 | Primary lesion and paired normal tissues | Surgery | ypT0 ypN0 |
|  | Patient 26 | Primary lesion and paired normal tissues | Surgery | ypT0 ypN0 |
|  | Patient 28 | Primary lesion and paired normal tissues | Surgery | ypT0 ypN0 |
|  | Patient 29 | Primary lesion and paired normal tissues | Surgery | ypT1 ypN0 |
|  | Patient 30 | Primary lesion and paired normal tissues | Surgery | ypT0 ypN0 |
|  | Patient 34 | Primary lesion and paired normal tissues | Surgery | ypT0 ypN0 |
|  | Patient 35 | Primary lesion and paired normal tissues | Surgery | ypT1 ypN0 |
|  | Patient 36 | Primary lesion and paired normal tissues | Surgery | ypT0 ypN0 |
|  | Patient 40 | Primary lesion and paired normal tissues | Surgery | ypT0 ypN0 |

scRNA-seq, single-cell RNA sequencing; scTCR-seq, single-cell T cell receptors sequencing; RNA-seq, RNA sequencing; CyTOF, cytometry by time-of-flight; WES, whole exome sequencing;

Table S4. Clinical characteristics at baseline of different set.

|  | **scRNA**  **(*n* = 2)** | **RNA-seq**  **(*n* = 11)** | **CyTOF**  **(*n* = 12)** | **WES**  **(*n* = 16)** |
| --- | --- | --- | --- | --- |
| **Median age (min-max), years** | 46.5 (39 - 54) | 46 (34 - 69) | 46.75 (34 - 69) | 44.94 (16 - 69) |
| **Females, n (%)** | 2 (100%) | 11 (100%) | 12 (100%) | 16 (100%) |
| **Menopausal status, n (%)** |  |  |  |  |
| Premenopausal | 2 (100%) | 8 (72.73%) | 7 (58.33%) | 10 (62.5%) |
| Perimenopausal | 0 (0%) | 1 (9.09%) | 1 (8.33%) | 1 (6.25%) |
| Postmenopausal | 0 (0%) | 2 (18.18%) | 4 (33.33%) | 5 (31.25%) |
| **Median Ki67 (min-max)** | 55 (40 - 70) | 58.18 (20 - 80) | 53.33 (20 - 75) | 59.38 (20 - 80) |
| **T stage, n (%)** |  |  |  |  |
| T1 | 1 (50%) | 0 (0%) | 1 (8.33%) | 1 (6.25%) |
| T2 | 1 (50%) | 9 (81.82%) | 10 (83.33%) | 12 (75%) |
| T3 | 0 (0%) | 0 (0%) | 1 (8.33%) | 2 (12.5%) |
| T4 | 0 (0%) | 2 (18.18%) | 0 (0%) | 1 (6.25%) |
| **N stage, n (%)** |  |  |  |  |
| N0 | 0 (0%) | 2 (18.18%) | 1 (8.33%) | 4 (25%) |
| N1 | 2 (100%) | 5 (45.45%) | 8 (66.67%) | 8 (50%) |
| N2 | 0 (0%) | 4 (36.36%) | 3 (25%) | 4 (25%) |
| **AJCC stage, n (%)** |  |  |  |  |
| IIA | 1 (50%) | 2 (18.18%) | 1 (8.33%) | 5 (31.25%) |
| IIB | 1 (50%) | 4 (36.36%) | 7 (58.33%) | 5 (31.25%) |
| IIIA | 0 (0%) | 3 (27.27%) | 4 (33.33%) | 5 (31.25%) |
| IIIB | 0 (0%) | 2 (18.18%) | 0 (0%) | 1 (6.25%) |
| **CPS, n (%)** |  |  |  |  |
| < 1 | 0 (0%) | 1 (9.09%) | 1 (8.33%) | 2 (12.5%) |
| ≥ 1, < 10 | 1 (50%) | 4 (36.36%) | 3 (25%) | 6 (37.5%) |
| ≥ 10, ≤ 100 | 1 (50%) | 6 (54.55%) | 8 (66.67%) | 8 (50%) |
| Unknown | 0 (0%) | 0 (0%) | 0 (0%) | 0 (0%) |
| **TPS, n (%)** |  |  |  |  |
| < 1% | 1 (50%) | 3 (27.27%) | 1 (8.33%) | 6 (37.5%) |
| ≥ 1%, < 50% | 1 (50%) | 4 (36.36%) | 7 (58.33%) | 7 (43.75%) |
| ≥ 50% | 0 (0%) | 1 (9.09%) | 2 (16.67%) | 1 (6.25%) |
| Unknown | 0 (0%) | 3 (27.27%) | 2 (16.67%) | 2 (12.5%) |

Data cutoff: June 30, 2023. scRNA-seq, single-cell RNA sequencing; scTCR-seq, single-cell T cell receptors sequencing; RNA-seq, RNA sequencing; CyTOF, cytometry by time-of-flight; WES, whole exome sequencing; AJCC, American Joint Committee on Cancer; CPS, combined positive score; TPS, tumor proportion score. PD-L1 status was evaluated by CPS and TPS using PD-L1 IHC 22C3 PharmDx.
