# Supplementary material for: Efficacy, safety and exploratory analysis of neoadjuvant tislelizumab (a PD-1 inhibitor) plus nab-paclitaxel followed by epirubicin/cyclophosphamide for triple-negative breast cancer: a phase 2 TREND trial
Source: Signal Transduct Target Ther. 2025 May 26;10:169. doi: 10.1038/s41392-025-02254-3 (PMC12104340; doi:10.1038/s41392-025-02254-3)
Supplement: Supplementary file 2 — study protocol [file 41392_2025_2254_MOESM2_ESM.pdf]

Clinical Study Protocol

**Study Title:**	A prospective, single-arm phase II clinical study on Tislelizumab in combination with albumin-bound paclitaxel followed by anthracycline and cyclophosphamide in the neoadjuvant treatment for triple-negative breast cancer (TREND study)
**Protocol Number:**	TREND
**Version No. and Version Date:**	Version 2.0/May 10, 2020
**Name of Medication:**	Baizean ® (Tislelizumab Injection)
**Study Phase:**	Phase II
**Sponsor:**	Liaoning Cancer Hospital & Institute
**Principal Investigator:**	Name: Zhang Qiang; Title: Chief Physician<br>Site: Liaoning Cancer Hospital & Institute<br>Address: No. 44, Xiaoheyan Road, Dadong District, Shenyang, Liaoning<br>Phone Number: +86-18900917799

Protocol Summary

**Study Title**	A prospective, single-arm phase II clinical study on Tislelizumab in combination with albumin-bound paclitaxel followed by anthracycline and cyclophosphamide in the neoadjuvant treatment for triple-negative breast cancer (TREND study)
**Investigational Drug**	Tislelizumab
**Study Population**	Patients with triple-negative breast cancer
**Name of Sponsor/Company**	Liaoning Cancer Hospital & Institute
**Protocol No.**	TREND
**Version Number/Date**	Version 2.0/May 20, 2020
**Study Duration:** The study of each subject will last from the treatment with Tislelizumab to surgery.	**Study Phase:** Phase II

Study Objectives:

Primary Objective:

To explore the pCR (pathologic complete response) rate (ypT0/Tis ypN0) after the addition of Tislelizumab for neoadjuvant chemotherapy based on standard albumin-bound paclitaxel followed by anthracycline chemotherapy

Secondary Objectives:

1. Objective response rate (ORR)
2. Safety

exploratory endpoints:

The correlations between efficacy and PD-L1 status, immune status of TME, and/or genetic profile.

Study Design:

Background: Triple-negative breast cancer (TNBC) approximately accounts for 15 to 20% of all breast cancers and has the characteristics of poor differentiation, high invasiveness, and early metastasis. Due to the lack of specific therapeutic targets, chemotherapy is still the main treatment strategy for TNBC. Therefore, the clinical treatment of TNBC is rather difficult. However, immunotherapy has made it hopeful to triumph over TNBC. The IMpassion130 study has demonstrated for the first time that Atezolizumab can significantly improve the progression-free survival (PFS) and overall survival (OS) of patients with advanced TNBC in first-line treatment. The KEYNOTE-012 and 086 studies have revealed that Pembrolizumab is effective as a first-line monotherapy in the treatment of advanced TNBC. Tislelizumab is a new type of PD-1 inhibitor with high specificity and affinity to the target point PD-1. Compared with Pembrolizumab, Tislelizumab has a higher affinity to PD-1 and a lower dissociation rate from PD-1. Theoretically, its efficacy is better. At present, clinical studies have been conducted on Tislelizumab in the treatment of such cancers as classic Hodgkin's lymphoma (CHL) and urothelial carcinoma, and positive results have been obtained. However, it has not yet been widely applied to the treatment of breast cancer.

Objective: To explore the efficacy and safety of Tislelizumab for the neoadjuvant therapy of TNBC based on albumin-bound paclitaxel followed by anthracycline and cyclophosphamide.

Method: This is a prospective, single-arm clinical study, in which 65 patients preliminarily diagnosed with TNBC by puncture immunohistochemistry at Liaoning Cancer Hospital & Institute from Nov 2020 to June 2023 are intended to be enrolled. They will receive Tislelizumab immunotherapy in combination with albumin-bound paclitaxel followed by anthracycline and cyclophosphamide. After eight cycles, surgery will be performed on them. The primary endpoint is pCR. The secondary endpoints include ORR, safety. The exploratory endpoints included potential correlations between efficacy and PD-L1 status, immune status of TME, and/or genetic profile.

Intended results: Tislelizumab can improve the pCR rate of the overall population with TNBC and has good safety. The efficacy of neoadjuvant chemotherapy can be predicted from the immune status of TME and PD-L1 status.

**Intended Number of Subjects**	A total of 65 patients are intended to be enrolled in the study.
**Inclusion Criteria**	1. Patients aged 18 to 70 years old;<br>2. Patients pathologically diagnosed with TNBC;<br>3. Patients newly diagnosed with treatment-naive non-metastatic breast cancer as determined through imaging evaluation;<br>4. Patients with T ≥ 2 cm or LN (+)<br>5. Patients with an ECOG score of 0 - 1;<br>6. Patients with good organ function (blood cells, heart, liver, kidney, and coagulation);

	7. Patients who have given informed consent and signed a written informed consent form (ICF);<br>8. Patients who have good compliance and are willing to receive follow-up, treatment, laboratory examinations, and other study procedures as planned.
**Exclusion Criteria**	1. Patients who have developed secondary primary malignant tumors in recent 5 years except for the fully treated skin basal cell carcinoma or cervical carcinoma in situ (CCIS);<br>2. Patients who have received chemotherapy, targeted therapy, and radiotherapy in the past 12 months;<br>3. Patients who have been treated with anti-PD-1, anti-PD-L1, or anti-PD-L2 drugs;<br>4. Patients who have developed active autoimmune diseases requiring systemic treatment in the past 2 years;<br>5. Patients who have been diagnosed with immunodeficiency or received immunosuppressive treatment within one week;<br>6. Patients with a medical history of human immunodeficiency virus (HIV) infection;<br>7. Patients with a medical history of non-infectious pneumonia treated with glucocorticoid;<br>8. Patients who currently suffer from pneumonia, active tuberculosis, active hepatitis B virus (HBV), or hepatitis C virus (HCV) infection;<br>9. Patients who are undergoing systemic treatment for active infection;<br>10. Patients who are currently participating in other clinical trials;<br>11. Patients who are pregnant or lactating women or are fertile but have not taken contraceptive measures;<br>12. Patients who are not suitable for participation in the study as determined by investigators because they have other serious acute/chronic physical/mental problems or abnormal laboratory examinations that may increase the risk related to the participation in the study or use of the drug or interfere with the determination of the study results.
**Investigational Drug, Strength, and Administration Method**	Drug name: Tislelizumab<br>Strength: 100 mg<br>Administration method: Patients will be first given albumin-bound paclitaxel 100 mg/m^2 weekly for 12 weeks. Epirubicin combined with cyclophosphamide will be given for 4 cycles at a dose of 75 mg/m^2 for epirubicin and 600 mg/m^2 for cyclophosphamide. Every 21 days is a cycle, and 200 mg of Tislelizumab immunotherapy will be given in the first day of each cycle. Patients will receive surgical treatment [breast preservation or mastectomy, sentinel lymph node biopsy (SLNB) or axillary lymph node dissection (ALND)] 2 to 6 weeks after the end of the last cycle of neoadjuvant therapy.

Efficacy Evaluation:

Primary efficacy indicators:

pCR rate (ypT0/Tis ypN0)

Secondary efficacy indicator:

1. ORR
2. Safety

Exploratory indicator:

The correlations between efficacy and PD-L1 status, immune status of TME, and/or genetic profile.

Statistical Methods:

The statistical description method will be mainly adopted. For measurement data, the mean, standard deviation, median, maximum, and minimum will be listed; for enumeration data and ranked data, the frequency (constituent ratio), rate and confidence interval will be listed. SPSS 23.0 Statistical Analysis Software will be used for programming calculation for all statistical analyses. The two-sided test will be used for all statistical tests. If $P \leq 0.05$, the differences tested will be considered statistically significant, and a 95% confidence interval will be used.

The measurement data of subjects in each visit will be statistically described using mean ± standard deviation or median (minimum, maximum). The paired t-test will be used to compare differences before and after treatment. The measurement

data of subjects in each visit will be statistically described using frequency (constituent ratio). Chi-square test, Fisher's precision probability test or non-parametric test will be used for changes before and after treatment.

Safety Indicator:

Safety will be evaluated by summarizing adverse events (AEs), changes in laboratory examination results, and changes in physical examination/vital signs. Any enrolled patients who have taken the investigational drug at least once will be included in the safety analysis.

Final analysis of study shall be based on data collected in the whole study period.

Contents

1. Overview **6**

2. Study Objectives and Endpoints **8**

2.1. Study objectives 8

2.1.1. Primary objectives 8

2.1.2. Secondary objectives 8

2.1.3. Exploratory objectives 8

2.2. Study endpoints 8

2.2.1. Primary endpoints 8

2.2.2. Secondary endpoints 8

2.2.3. Exploratory endpoints 9

3. Study Design **9**

3.1. Overall design 9

3.2. Study subject 9

3.2.1. Inclusion criteria 10

3.2.2. Exclusion criteria 10

3.2.3. Stratification factors 10

3.3. Screening period 11

3.4. Treatment period 11

3.4.1. Neoadjuvant therapy period 11

3.4.1.1 Treatment 11

3.4.1.2 Clinical evaluation 11

3.4.1.3 Pathological evaluation 12

3.4.1.4 Sample collection 12

3.4.2. Radical mastectomy 11

3.4.3. Radiation therapy 12

3.5. Early termination visit and safety follow-up 12

3.6. Long-term follow-up 12

3.7. Survival follow-up 13

3.8. Discontinuation of study treatment or study 13

3.8.1. Patient discontinuation of study treatment 13

3.8.2. Patient discontinuation of study (End of study for individual patient) 14

3.9. End of study 14

4. Statistical Analysis Methods **14**

4.1. Data quality assurance 14

4.2. General considerations 15

4.2.1. General analytical methods 15

4.2.2. Relevant definitions 15

4.2.3. Analysis window 17

4.2.4. Missing data and outliers 17

4.2.5. Analysis software 17

4.3. Study subjects 17

4.3.1. Analysis dataset 17

4.3.2. Subject disposition 17

4.3.3. Protocol deviations 17

4.4. Demographic and baseline characteristics 17

4.5. Previous and concomitant treatment 18

4.6. Treatment compliance 18

4.7. Tumor assessment 18

4.8. Analysis of efficacy endpoints ... 18
4.8.1. pCR rate (ypT0/Tis ypN0 criteria) ... 18
4.8.2. Objective response rate (ORR)... 19
4.8.3. TILs and CPS subgroup pCR rate (ypT0/Tis ypN0 criteria)... 19
4.8.4. Event-free survival (EFS)... 19
4.8.5. Overall survival (OS) ... 20
4.9. Safety analysis... 20
4.9.1. Drug exposure ... 20
4.9.2. Adverse event... 21
4.9.3. Laboratory test... 22
4.9.4. Vital signs and weight ... 22
4.9.5. ECOG performance status scores... 22
4.9.6. 12-lead ECG... 22
4.9.7. Echocardiogram/MUGA ... 22
4.10. Interim analysis ... 23
4.11. Exploratory analysis ... 23
4.12. Sample size consideration ... 23
5. References... 23
Appendix 1 Schedule of Assessments... 24
Appendix 2 Clinical Laboratory Assessment... 29
Appendix 3 ECOG performance status scores ... 31
Appendix 4 Judgment Rules for Concomitant Medication ... 32
Appendix 5 Judgment Rules for TRAEs... 33
Appendix 6 Imputation Rules for Date of Death... 34
Appendix 7 Last Day Known to be Live... 35

Overview

This is a prospective, single-arm Phase II clinical study to evaluate the efficacy and safety of tislelizumab combined with nab-paclitaxel and anthracycline chemotherapy in neoadjuvant therapy for TNBC. Potential biomarkers associated with efficacy, tolerability, and/or progressive disease (PD) in tumor tissue and peripheral blood of TNBC received immune therapy will also be explored.

The St Gallen 2011 Consensus divides breast cancer into four subtypes, in which estrogen (ER) receptor-negative, progesterone (PR) receptor-negative, and HER-2 negative breast cancer are defined as triple-negative breast cancer (TNBC) with an incidence of 15-20%. Compared with hormone receptor-positive or HER-2 positive breast cancer, TNBC is highly aggressive, and has an early age of onset, a greater risk of metastasis, and a poor prognosis. So far, there is no specific target drug for TNBC, and chemotherapy is still the standard treatment. And neoadjuvant chemotherapy is currently the preferred strategy for TNBC with a certain tumor burden. Neoadjuvant chemotherapy can not only provide the possibility of surgery for inoperable patients, but also improve the breast conservation rate. Patients who achieve pathological complete response after neoadjuvant chemotherapy can achieve long-term disease-free survival and overall survival, while patients who fail to achieve pathological complete response can receive adjuvant intensive therapy. Currently, in neoadjuvant therapies for early TNBC patients, the relevant guidelines support the use of pathological complete response (pCR) rate as a clinical study endpoint.

Solvent-based paclitaxel is one of the most commonly used drugs in neoadjuvant chemotherapy, but both the drug itself and the solvent can cause the corresponding toxic and side reactions. Compared with solvent-based paclitaxel, nab-paclitaxel, as a new solvent-free paclitaxel, avoids hypersensitivity reactions caused by co-solvent, does not require hormone pretreatment, shortens the infusion time, and features target enrichment in tumor tissues. The GeparSepto-GBG 69 study showed that nab-paclitaxel replacing solvent-based paclitaxel as neoadjuvant chemotherapy significantly increased the pCR rate in the total population from 29% to 38%. The increase was more prominent in the TNBC subtype, with an absolute count of 22%, showing its advantage in TNBC.

Immunosuppressant monoclonal antibodies against programmed cell death protein ligand 1 (PD-L1) have been shown to have significant antitumor effects in metastatic TNBC with low toxic and side reactions, and the efficacy is particularly significantly in first-line treatment subgroups. Chemotherapy can increase the release of tumor-specific antigens, and on this basis, while immune checkpoint inhibitors (ICIs) may enhance endogenous anti-tumor immune activity. Therefore, the Phase I KEYNOTE-173 trial and the Phase II I-SPY2 trial explored the efficacy of chemotherapy combined with ICI, and both obtained positive results. In HER2-negative TNBC patients, the pCR rate for pembrolizumab combined with neoadjuvant chemotherapy was higher than that for neoadjuvant chemotherapy alone. The Phase III clinical study KEYNOTE-522 once again confirmed the efficacy of pembrolizumab in neoadjuvant therapy for TNBC. Pembrolizumab not only improved the pCR rate, but also extended the relapse-free survival of patients.

In summary, both nab-paclitaxel and ICIs can achieve very good efficacy in TNBC treatment. Will the combination of nap-paclitaxel and ICIs bring more benefits? The IMpassion130 study was the first trial of nap-paclitaxel combined with ICI in TNBC. The study showed that nap-paclitaxel combined with atezolizumab significantly improved the progression-free survival compared with nap-paclitaxel alone, and the overall survival (OS) was also extended in the PD-L1-positive subgroup. This study has brought good news for the treatment of advanced TNBC patients, but there are few clinical studies of nap-paclitaxel combined with ICIs for neoadjuvant chemotherapy in the world.

Tislelizumab is a new PD-1 inhibitor developed by BeiGene. It is a humanized IgG4 mutant monoclonal antibody with high specificity and affinity for its target PD-1. Compared with pembrolizumab, tislelizumab

has a higher affinity to PD-1 and a lower dissociation rate from PD-1. Theoretically, its efficacy is better. At present, tislelizumab has been clinically studied in urothelial carcinoma with positive results, and has not been involved in other cancer types. Therefore, the objective of this study is to investigate the efficacy and safety of add-on tislelizumab on the basis of nab-paclitaxel combined with anthracycline chemotherapy in neoadjuvant therapy for TNBC.

Study Objectives and Endpoints

Study objectives

Primary objectives

To evaluate the pCR rate in all patients with TNBC treated with tislelizumab in combination with nab-paclitaxel and anthracycline chemotherapy by pathologists according to ypT0/Tis ypN0 criteria at breast surgery.

Secondary objectives

To evaluate the objective response rate (ORR) of tislelizumab combined with nab-paclitaxel and anthracycline chemotherapy in TNBC patients in the neoadjuvant period by the investigator according to RECIST Version 1.1;

To evaluate the safety and tolerability of tislelizumab combined with nab-paclitaxel and anthracycline neoadjuvant therapy in TNBC patients by the investigator according to NCI-CTCAE v5.0;

Exploratory objectives

To explore potential biomarkers associated with efficacy, tolerability, and/or progressive disease (PD) in tumor tissue and peripheral blood;

Study endpoints

Primary endpoints

pCR rate of all patients evaluated by pathologists using ypT0/Tis ypN0 criteria at radical mastectomy;

- pCR rate (ypT0/Tis ypN0): defined as the percentage of subjects with no residual invasive cancer in completely resected breast specimens and all sampled regional lymph nodes evaluated by hematoxylin and eosin staining by pathologists using the American Joint Committee on Cancer (AJCC) staging criteria at radical mastectomy following completion of neoadjuvant therapy.

Secondary endpoints

To evaluate the objective response rate (ORR) of tislelizumab combined with nab-paclitaxel and anthracycline chemotherapy in TNBC patients in the neoadjuvant setting by the investigator according to RECIST Version 1.1;

- ORR: Defined as the proportion of patients who achieve a 30% decrease in the sum of diameters of target lesions and maintain the reduction for the required minimum time limit according to RECIST Version 1.1 from the baseline before the first neoadjuvant therapy to the time of surgery. ORR is a short-term efficacy evaluation index and is the sum of complete response (CR) rate and partial response (PR) rate.
- CR: Complete Response, defined as disappearance of all target lesions. Any pathological lymph nodes (whether target or non-target) must have reduction in short axis to <10 mm.
- PR: Partial Response, defined as at least a 30% decrease in the sum of diameters of target lesions, taking as reference the baseline sum diameters.
- Progressive Disease (PD): At least a 20% increase in the sum of diameters of target lesions, taking as reference the smallest sum on study (this includes the baseline sum if that is the smallest on study). In addition to the relative increase of 20%, the sum must also demonstrate an absolute

increase of at least 5 mm. (Note: the appearance of one or more new lesions is also considered progression).

- Stable Disease (SD): Neither sufficient shrinkage to qualify for PR nor sufficient increase to qualify for PD, taking as reference the smallest sum diameters while on study.

Safety and tolerability of tislelizumab combined with nab-paclitaxel and anthracycline neoadjuvant therapy in TNBC patients evaluated by the investigator according to the incidence, nature, and severity of AEs and serious AEs (SAEs) per NCI-CTCAE v5.0, clinical laboratory abnormalities, relevant physical examination, ECG, and vital signs, and the percentage of subjects who discontinued study treatment due to AEs;

Exploratory endpoints

Status of exploratory biomarkers in archived and/or freshly collected tumor tissue and collected blood (or blood derivatives), including but not limited to, PD-L1 expression, immune status of TME, and immune-related gene expression profiles, and their association with disease status and/or response to neoadjuvant tislelizumab in combination with chemotherapy.

Study Design

Overall design

This is a prospective, single-center, single-arm Phase II clinical study in patients with TNBC. Neoadjuvant therapy will be administered every 3 weeks until any of the following occurs (whichever occurs first): 1) completion of 8-cycle treatment; 2) intolerable toxicity; 3) disease progression (based on RECIST V1.1); 4) withdrawal of informed consent, loss to follow-up, or death.

Figure 1: Study Diagram

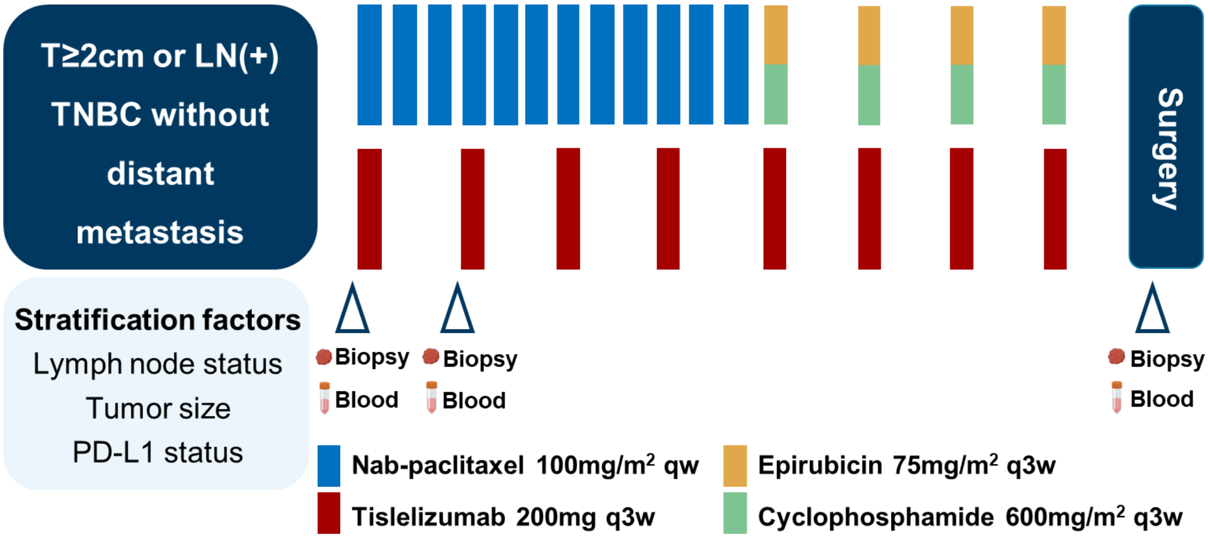

Abbreviations: LN= lymph node; TNBC= triple-negative breast cancer; pCR= pathological complete response; PD-L1= programmed cell death protein ligand-1; TILs= tumor infiltrating lymphocytes; qW= once a week; q3W= once every 3 weeks;

Study Subjects

This is a prospective, single-arm phase II clinical study, in which 65 patients preliminarily diagnosed with TNBC by puncture immunohistochemistry at Liaoning Cancer Hospital & Institute from May 2020 to May 2023 are intended to be enrolled.

Inclusion Criteria

1. Patients aged 18 to 70 years old;
2. Patients pathologically diagnosed with TNBC;
3. Patients newly diagnosed with treatment-naive non-metastatic breast cancer as determined through imaging evaluation;
4. Patients with T ≥ 2 cm or LN (+)
5. Patients with an ECOG score of 0 - 1;
6. Patients with good organ function (blood cells, heart, liver, kidney, and coagulation);
7. Patients who have given informed consent and signed the informed consent form (ICF);
8. Patients who have good compliance and are willing to receive follow-up, treatment, laboratory examinations, and other study procedures as planned.

Exclusion Criteria

1. Patients who have developed secondary primary malignant tumors in recent 5 years except for the fully treated skin basal cell carcinoma or cervical carcinoma in situ (CCIS);
2. Patients who have received chemotherapy, targeted therapy, and radiotherapy in the past 12 months;
3. Patients who have been treated with anti-PD-1, anti-PD-L1, or anti-PD-L2 drugs;
4. Patients who have developed active autoimmune diseases requiring systemic treatment in the past 2 years;
5. Patients who have been diagnosed with immunodeficiency or received immunosuppressive treatment within one week;
6. Patients with a medical history of human immunodeficiency virus (HIV) infection;
7. Patients with a medical history of non-infectious pneumonia treated with glucocorticoid;
8. Patients who currently suffer from pneumonia, active tuberculosis, active hepatitis B virus (HBV), or hepatitis C virus (HCV) infection;
9. Patients who are undergoing systemic treatment for active infection;
10. Patients who are currently participating in other clinical trials;
11. Patients who are pregnant or lactating women or are fertile but have not taken contraceptive measures;
12. Patients who are not suitable for participation in the study as determined by investigators because they have other serious acute/chronic physical/mental problems or abnormal laboratory examinations that may increase the risk related to the participation in the study or use of the drug or interfere with the determination of the study results.

Stratification Factors

PD-L1 positive vs PD-L1 negative

Lymph node positive vs lymph node negative

Tumor size

Screening period

The screening assessment will be performed within 28 days prior to the first dose of the investigational drug. Patients who agree to participate will sign an informed consent form (ICF) prior to screening procedures. Tumor tissue must be collected at baseline for biomarker analysis and, in the judgment of the investigator, be able to undergo radical mastectomy or breast-conserving surgery for affected breasts, and sentinel lymph node biopsy or lymph node dissection for axillary lymph nodes following neoadjuvant therapy. Patients will be assessed for eligibility by the investigator based on the latest screening assessment results.

Treatment period

Subjects may receive other medications deemed medically necessary by the investigator. Discontinuation of study treatment may be required if there are clinical indications for any drug or vaccine specifically prohibited during the trial.

Neoadjuvant therapy period

Treatment

After completing all screening activities, patients confirmed by the investigator as eligible will be enrolled to receive neoadjuvant therapy with tislelizumab on the basis of standard nab-paclitaxel combined with anthracycline therapy. Neoadjuvant therapy is administered for a maximum of 8 cycles. Investigational drug, dose and route of administration were as following:

- Tislelizumab, 200 mg, is administered as an intravenous infusion on Day 1 of each 21-day cycle for a total of 8 cycles. If tislelizumab is administered on the same day as chemotherapy, tislelizumab should be infused first.
- Nab-paclitaxel, 100 mg/m^2, will be administered by intravenous infusion in 21-day cycles on Days 1, 8, and 15 of the first 4 cycles, for a total of 4 cycles. When nab-paclitaxel is administered on the same day as Tislelizumab, nab-paclitaxel should be infused after Tislelizumab.
- Epirubicin, 75 mg/m^2, will be administered by intravenous infusion in 21-day cycles on Day 1 of the last 4 cycles, for a total of 4 cycles.
- Cyclophosphamide, 600 mg/m^2, will be administered by intravenous infusion in 21-day cycles on Day 1 of the last 4 cycles, for a total of 4 cycles.

Neoadjuvant therapy is administered every 3 weeks until any of the following occurs (whichever occurs first):

- They should complete 8 cycles of treatment;
- Intolerable toxicity;
- Disease progression (based on RECIST 1.1);
- Withdrawal of informed consent, loss to follow-up, or death;

Tumors are assessed every 6 weeks (± 7 days) and subsequent therapy is administered at the investigator's discretion if a patient develops PD.

If treatment is delayed, all assessment procedures should be performed according to the new dosing schedule.

Clinical evaluation

All patients will receive a breast MRI examination every 2 cycles to evaluate neoadjuvant efficacy (based on RECIST 1.1). In addition, ultrasound, blood routine, liver and kidney function tests, and lymphocyte

immunoassay will be conducted once every cycle. After the first cycle or once disease progression, a puncture pathological examination will be performed to evaluate changes in drug resistance and tumor microenvironment, etc. Adverse reactions will be evaluated according to the National Cancer Institute's Common Terminology Criteria for Adverse Events (CTCAE 5.0).

Pathological evaluation

The tumor tissues obtained by puncture and surgery will be stained with HE in the pathology department of Liaoning Cancer Hospital & Institute. After the neoadjuvant chemotherapy is completed, the pCR rate will be evaluated according to the definition of the pathological stage of ypT0/Tis ypN0 (after neoadjuvant chemotherapy (yp for short)).

Sample collection

Tumor samples were collected from biopsies obtained from at least one tumor site before administration of the first therapy dose, after cycle one and at the time of surgery.

PD-L1 IHC 22C3 pharmDx assay will be conducted for the tumor tissues obtained by puncture in the central laboratory of Liaoning Cancer Hospital & Institute. CPS will be used to define PD-L1 expression, which is the number of PD-L1 positive cells (tumor cells, lymphocytes and macrophages) divided by the total number of tumor cells multiplied by 100. If CPS is >1, PD-L1 expression will be considered positive.

Blood samples were collected at screening, every week upon treatment, and at the end of treatment or disease progression.

Radical mastectomy

Each subject will undergo radical mastectomy 2 to 6 weeks after the last cycle of neoadjuvant therapy. Radical mastectomy will be performed according to local standards of care, including breast conserving surgery or mastectomy with axillary sentinel lymph node biopsy or axillary lymph node dissection.

Radiation therapy

Postoperative radiation therapy is acceptable according to applicable standards of care, for example, in the case of breast conserving surgery, large primary tumor, presence of lymph node metastases.

Early termination visit and safety follow-up

Patients who discontinue treatment for any reason should return to the site for an early termination visit within 30 days after the last dose of the investigational drug or before the start of new anti-cancer therapy, whichever comes first. If routine laboratory tests (e.g., hematology, clinical chemistry) are performed within 7 days prior to the early termination visit, they do not need to be repeated. If the previous tumor assessment is performed within 6 weeks prior to the early termination visit, it does not need to be repeated at the early termination visit. If the investigational drug is originally interrupted due to AE and then permanently discontinued, the early termination visit can follow.

Patients should be followed for AEs regardless of whether they start new anti-cancer therapy, including: 1) chemotherapy-related AEs until 30 ± 7 days after the last dose; 2) immune-drug-related AEs until 90 ± 7 days after the last dose of tislelizumab; 3) surgery-related AEs until 30 ± 7 days after the surgery. Subjects with AEs > Grade 1 will be followed until the AE is resolved to Grade 0-1.

Long-term follow-up

Subjects who discontinue study treatment in the neoadjuvant therapy period for any reason other than disease progression and are unable to undergo definitive surgery (defined as local and/or distant disease progression)

will enter the long-term follow-up period and should be assessed for pCR (defined as non-pCR for those who have not undergone surgery) and disease progression/recurrence. Date of disease recurrence, progression, start and end dates of subsequent anti-cancer therapy, and reason for treatment should be recorded on the appropriate eCRF. Subjects who discontinue study treatment and continue to receive another neoadjuvant therapy prior to definitive surgery will be available for pCR and/or imaging evaluation.

In-office long-term follow-up assessment is performed every 3 months (± 1 month) after adjuvant therapy to assess recurrent or metastatic disease. Other tests/imaging assessments (e.g., bone/liver scan) for recurrent or metastatic disease will be performed at the discretion of the subject's treating physician according to local standard of care or at the onset of symptoms. Unless a patient requests withdrawal from follow-up, all patients will be followed by the site for survival status and subsequent anti-cancer therapy. Long-term follow-up ends at 3 years after initiation of drug therapy.

Additional tests/imaging assessments (e.g., bone/liver scan) for recurrent or metastatic disease will be performed at the discretion of the subject's treating physician according to local standard of care or at the onset of symptoms. The trial procedures performed at each visit are summarised in the trial flow chart in Appendix 1.

If a subject does not return for protocol-specified visit assessments during the long-term follow-up period, every effort should be made to reach the subject by telephone every 6 months (± 1 month) for survival status.

Survival follow-up

If a subject is withdrawn from treatment due to recurrent or metastatic disease at any time during the neoadjuvant therapy or long-term follow-up periods, survival follow-up information should be collected by telephone, patient medical records, and/or clinical visits every 6 months (± 1 month) until death, loss to follow-up, withdrawal of informed consent, or study termination by the sponsor, whichever occurs first. For subjects who die during the follow-up period, the date and cause of death should be recorded on the appropriate eCRF.

Discontinuation of study treatment or study

Patient discontinuation of study treatment

Patients have the right to discontinue study treatment at any time for any reason. In addition, the investigator has the right to discontinue the study treatment for any patient at any time. Discontinuation of treatment does not mean withdrawal from the trial and patients who discontinue study treatment should be followed up if possible and the primary reason for discontinuation should be documented on the appropriate electronic case report form (eCRF). Patients may discontinue study treatment for reasons including but not limited to:

- Withdrawal of ICF by the subject
- Pregnancy
- Intolerable adverse events
- A medical condition may jeopardize the patient's safety at the discretion of the investigator if the patient continues the study treatment;
- Delayed operation time by the judgment of investigator, including but not limited to: in neoadjuvant therapy period, if disease progression occurs at the scheduled tumor assessment or at any other time point, radical breast cancer surgery is not performed 2 weeks after assessment of disease progression without justification; those who complete or fail to complete 6 cycles of neoadjuvant

therapy due to AE (the total duration of neoadjuvant therapy is not more than 21 weeks) do not undergo radical breast cancer surgery within 6 weeks after the end of treatment without justification;

- Use of any other antineoplastic therapy (i.e., chemotherapy, hormonal therapy, immunotherapy, or standard agents or investigational agents for the treatment of cancer [including Chinese herbal medicine and Chinese patent medicine])
- Patient non-compliance with treatment regimen
- Progressive Disease

Patient discontinuation of study (End of study for individual patient)

Patients may discontinue the study for reasons including but not limited to:

- Withdrawal of ICF by the subject
- Death
- Lost to follow up
- Completion of all study assessments
- Termination of the study by the sponsor

End of study

End of study is defined as the date of the last patient last visit (LPLV) or the date of the last data point required to collect statistical analysis or safety follow-up for the last patient, whichever occurs later.

The leading site has the right to terminate this study at any time. Reasons for early termination may include, but are not limited to:

- The incidence or severity of an AE in this or other studies indicates a potential health hazard to subjects
- Overall patient enrollment is unsatisfactory and recruitment is too slow
- When 48 subjects enrolled, $\leq$ 20 valid cases achieve pCR rate (ypT0/Tis ypN0)

If the decision is made to terminate the study, the leading site will notify each investigator. The leading site will notify the sponsor. Patients who prematurely discontinue treatment have to undergo EOT visit as soon as possible.

The sponsor will be responsible for informing early termination of the study to the Institutional Review Board (IRB)/Independent Ethics Committee (IEC) to fully consider and protect the interests of the patient.

Statistical Analysis Methods

Data quality assurance

According to ICH GCP guidelines, the study director has direct access to source documents to verify the consistency of data recorded in the CRF.

The CRF should be routinely reviewed at regular intervals throughout the study by the study director or his/her designee to verify compliance with the study protocol and completeness, consistency, and accuracy of the data entered. In order to ensure that all issues identified during monitoring visits are resolved, all investigators will work with the study director.

All tables, figures and data listings to be included in the statistical analysis report will be independently checked according to the standard operating procedures to ensure their consistency and completeness.

General considerations

General analytical methods

(1) Descriptive statistics

Unless otherwise specified, the following summary of descriptive statistics will be presented by type of variables:

- Continuous data will be summarized as mean, standard deviation (SD), median, minimum, maximum, and number of observations.
- Categorical data will be summarized as frequency counts and percentages based on the number of subjects at the corresponding time point. Change from baseline in categorical data will be summarized using cross-tabulations where appropriate.

(2) Decimal places

Unless otherwise specified, the number of decimal places of minimum and maximum values is consistent with that recorded on the CRF; the number of decimal places of mean and median is 1 more than that of the original data; the number of decimal places of standard deviation is 2 more than that of the original data; the percentage is retained to 1 decimal place; and the number of decimal places of all statistics is generally no more than 4.

P-values greater than or equal to 0.001 are retained to three decimal places, P-values less than 0.001 are presented as "< 0.001", and confidence intervals are presented to 1 more decimal place than the original data.

Relevant definitions

Baseline

Unless otherwise specified, "Baseline" is defined as the last non-empty test value prior to (inclusive) the first dose.

Day/Month/Year conversion

Month = Day/30.4375, Year = (Day)/365.25, rounded to one decimal place.

Previous and concomitant treatment

Previous therapies are defined as medications that stopped prior to the date of first dose of the investigational drug.

Concomitant therapies are defined as drug therapies or non-drug therapies that 1) start prior to the first dose of the investigational drug and are continuing at the first dose of the investigational drug, or 2) start on or after the date of the first dose of the investigational drug until 30 days after the patient's last adjuvant dose.

If the start date or end date of previous and concomitant therapy is missing, the rules for judgment are provided in Appendix 4.

Adverse events (AEs)

Adverse event (AE) refers to any unfavorable or unintended sign (including abnormal laboratory finding), symptom or disease (new or exacerbated) temporally associated with the use of an investigational drug, whether or not considered related to the investigational drug.

Only SAEs should be reported from the signing of informed consent until before investigational drug administration.

After initiation of the investigational drug, all AEs and SAEs, regardless of relationship to the investigational drug and surgery, are reported until 30 days after the last dose of chemotherapy agents, 90 days after the last dose of tislelizumab, or 30 days after radical breast cancer surgery, whichever occurs first.

The investigator should report all SAEs assessed as related to the investigational drug even after discontinuation of treatment.

Treatment-related adverse event (TRAE)

TRAE is defined as an AE that newly occurred or worsened in severity from baseline after the first dose of the investigational drug to 30 days after discontinuation of the investigational drug or the start of new anticancer therapy, whichever occurs first. TRAE are AEs that are considered to be related to the study treatment, indicating that the investigational drug or therapy may have contributed to the event.

If the start date or end date of an AE is missing, the rules for judgment are provided in Appendix 5.

Relationship of AEs

Investigational drug-related adverse events refer to “adverse events” in the EDC that are “related” for the “relationship with tislelizumab”, or “related” for the “relationship with nab-paclitaxel”, or “related” for the “relationship with epirubicin”, or “related” for the “relationship with cyclophosphamide”.

Procedure-related AEs refer to AEs categorized as "procedure-related AEs" in EDC "AEs".

Study follow up time

Follow-up time is defined as the number of days from the date of the first dose of the investigational drug to the subject's completion/withdrawal from the trial. Date of first dose is defined as D1 and there is no D0 follow-up date. Follow-up time equals 'Date of subject's completion/withdrawal' - Date of first dose of investigational drug + 1.

Note: "Date of completion/withdrawal" refers to data of "Date of Completion/Withdrawal" on EDC study summary page.

Study date

Study date is defined as the number of days from the date of the first dose of the investigational drug to the date of assessment. Date of first dose is defined as D1 and there is no D0 study date. If the assessment occurs before the date of the first dose, the study day equals to the date of assessment - date of first dose; if the assessment occurs after the date of the first dose, then the study day equals to the date of assessment - date of the first dose + 1.

Study duration

The neoadjuvant therapy period is defined as the date of the first neoadjuvant therapy until before the date of surgery for patients who underwent radical surgery or 30 days after the date of the last neoadjuvant therapy for patients who did not undergo surgery.

The surgical period is defined as the date of surgery until the date of the first adjuvant therapy or 30 days after surgery.

Analysis window

For post-baseline visits, statistical analyses will be performed at protocol-scheduled visit time points when performing analyses by visit, and unscheduled time points in the protocol are not considered. Protocol unscheduled test results will be presented in listings.

Missing data and outliers

Unless otherwise stated, all analyses will be based on observed data and imputation of missing data will not be considered; if there are outliers, the method of handling will be discussed and determined at the data review meeting. If there are values with ">", "<", "≤", or "≥" in the data, they are listed directly in the list, and when summarizing the analysis, the analysis is processed according to the value after removing the mathematical operators. Specific handling rules of missing dates are provided in Appendix 4-5.

Analysis software

All reported results will use SPSS V22, SAS® 9.4 or higher version in a secure and validated environment.

All report outputs will be provided to the sponsor in Microsoft Word [2019] document/RTF/PDF format.

Study subjects

Analysis dataset

Safe Analysis Set (SS): Subjects who have received at least one dose of any investigational drug (including nab-paclitaxel, epirubicin, cyclophosphamide, and tislelizumab) and have at least one safety evaluation.

The efficacy evaluable set (EES): All subjects who received neoadjuvant tislelizumab plus chemotherapy, underwent documented surgery, and had available pathological efficacy without major protocol deviation.

Subject disposition

The number and percentage of subjects who were screened, failed screening, enrolled, received at least one dose, discontinued study treatment, failed to undergo surgery, and terminated the study are summarized, along with the cutoff date and the status of subjects who entered survival follow-up. The primary reasons for study termination are analyzed, and the number and percentage of subjects included in the efficacy set are summarized.

Subject disposition flow chart is provided.

Subjects who failed screening/did not complete screening and subjects who terminated the study are listed separately.

Protocol deviations

Categories of major protocol deviations are summarized based on subjects who signed informed consent and a listing of all protocol deviations will be provided.

Criteria for major protocol deviations will be developed and patients with major protocol deviations will be identified and documented prior to database lock.

Demographic and baseline characteristics

The following demographic and baseline characteristics are statistically described and tabulated based on ES.

- Demographic information (age, gender, fertility, height, weight, BMI)

Age = INT ((Date of signing informed consent - Date of birth + 1)/365.25), INT is a rounding function.

- Baseline clinical characteristics (medical history, allergy history, history of non-tumor surgery, baseline vital signs, baseline physical examination, and baseline ECOG score)
- Tumor diagnosis (time to initial diagnosis of cancer, histopathological grade, clinical stage at initial diagnosis, TNM stage at initial diagnosis, baseline PD-L1 count)

Time to initial diagnosis of cancer (months) = 12*(Date of current diagnosis - Date of initial diagnosis)/365.25

- Prior tumor therapy (any prior chemotherapy for tumor, any prior radiation therapy for tumor, any prior targeted therapy for tumor, any prior surgery for tumor, any other prior tumor therapy)
- Screening for infectious disease (HBsAg, HBsAb, HBcAb, and HCV antibodies)

Previous and concomitant treatment

WHODrug Dictionary (September 2020) or its most recent version is used for previous and concomitant medications during the study.

MedDRA Chinese 23.1 or its latest version is used for concomitant non-drug therapies during the study.

Previous and concomitant medications are summarized by ATC quaternary classification and preferred name PN based on ES, and concomitant non-drug therapies are summarized by system organ class SOC and preferred term PT, and corresponding listings are provided.

Treatment compliance

Whether dosing is adjusted, type of adjustment and reason for adjustment are summarized separately for each cycle by investigational drug based on ES. The number of subjects who underwent radical breast cancer surgery, length of surgery, intraoperative estimated blood loss, intraoperative estimated blood transfusion volume, and intraoperative complications are summarized, and a list of surgical records is provided.

Tumor assessment

Tumor response is assessed by the investigator according to RECIST 1.1.

Based on ES, the overall tumor response assessment at each time point is summarized, the best overall response, objective response rate, and disease control rate are summarized, and a listing of tumor response assessments is provided.

Note: Objective response = complete response + partial response; Disease control = complete response + partial response + stable disease.

Analysis of efficacy endpoints

pCR rate (ypT0/Tis ypN0 criteria)

The primary study endpoint will be pCR rate (ypT0/Tis ypN0) in all patients assessed by pathologists at radical mastectomy using ypT0/Tis ypN0 criteria, and will be analyzed based on the EES. According to recent clinical studies, a pCR rate of 41% (all treated population, both surgical and non-surgical) is assumed for historical control (standard chemotherapy regimen). The expected pCR estimate for this study is 56% (all treated population, both surgical and non-surgical), representing clinically significant improvement. The resulting null and alternative hypotheses are as follows:

H0: pCR rate ≤ 41%

Ha: pCR rate > 41%

Based on the group sequential design of the binary endpoint of single-arm trials, an interim efficacy and safety analysis is scheduled for the trial after enrollment of 48 patients. If there are ≤ 20 responders for pCR (ypT0/Tis ypN0), the trial will be terminated for ineffectiveness. If there are ≥ 30 responders for pCR, the primary endpoint is reached and the trial can be early terminated. If the number of patients who achieved pCR was greater than or equal to 30, accrual could be terminated early after reaching the primary endpoint. This design yielded a one-sided type 1 error of 0.05 and a power of 80.0% when the true response rate was 56%. In this case, it can be concluded that tislelizumab combined with nab-paclitaxel and anthracycline neoadjuvant therapy can statistically significantly increase the pCR rate (ypT0/Tis ypN0) compared with historical controls, thus demonstrating the superiority of tislelizumab combined with nab-paclitaxel and anthracycline neoadjuvant therapy.

The number and percentage of subjects achieving pCR are summarized based on ES (both surgical and non-surgical), and Clopper-Pearson 95% confidence interval (CI) is constructed to provide the statistics and P values corresponding to the chi-square test and the corresponding listings are provided.

Objective response rate (ORR)

To evaluate the ORR of tislelizumab combined with nab-paclitaxel and anthracycline in TNBC patients during the neoadjuvant period by the investigator according to RECIST Version 1.1;

Based on the ES, the efficacy of neoadjuvant therapy in subjects will be evaluated according to RECIST Version 1.1. The number and percentage of subjects achieving CR, PR, SD and PD will be summarized, and the Clopper-Pearson 95% CI will be constructed; the corresponding statistics and P-values of Chi-square test will be provided, as well as the corresponding lists.

CPS subgroup pCR rate (ypT0/Tis ypN0 criteria)

The pCR rate and ORR of tislelizumab combined with nab-paclitaxel and anthracycline neoadjuvant chemotherapy in TNBC patients with different CPS and peripheral blood immune lymphocyte status will be evaluated by pathologists according to ypT0/Tis ypN0 criteria and RECIST V1.1 during radical breast cancer resection.

The number and percentage of subjects in each group achieving pCR are summarized based on CPS and peripheral blood immune lymphocyte status of subjects in ES (both surgical and non-surgical), and 95% CI is constructed to provide the corresponding statistics and the corresponding listings are provided.

Event-free survival (EFS)

EFS: defined as the time from the first neoadjuvant therapy to disease progression precluding surgery, local or distant recurrence, second primary malignancy (breast or other cancer), or death event from any cause.

Event-free curves and corresponding quantiles, including median, will be estimated using the Kaplan-Meier (KM) method based on ES. Brookmeyer and Crowley method (Brookmeyer and Crowley, 1982) is used if the two-sided 95% confidence interval of the median can be estimated. EFS rates at 1, 2, and 3 years from the first dose will be estimated using the KM method, and corresponding 95% confidence intervals will be estimated using Greenwood's formula (Greenwood, 1926).

EFS Censoring Rules

Description	Event/Censoring	Date
Disease progression precluding radical breast cancer surgery	Event	Clinical disease progression date
Local or distant recurrence	Event	Clinical disease progression date
Second primary malignancy	Event	Clinical disease progression date

Death from any cause	Event	Death date
New antineoplastic therapy	Censoring	Date of last tumor assessment before new antineoplastic therapy
No events as of cut-off date	Censoring	Date of last tumor assessment
Loss to follow-up for at least two tumor assessments or permanent loss to follow-up	Censoring	Date of last tumor assessment
Study termination for reasons other than those listed above (e.g., withdrawal of informed consent, major protocol deviation, etc.)	Censoring	Date of last tumor assessment

Overall survival (OS)

OS is defined as the time from the start of the first neoadjuvant therapy to death from any cause.

Based on ES, median OS, OS rates at 1, 2, and 3 years from the first dose will be calculated and presented with two-sided 95% CIs. The analysis method is the same as EFS.

OS Censoring Rules

Description	Event/Censoring	Date
Death from any cause	Event	Death date
Survival as of cutoff date	Censoring	Cutoff date
Lost to follow up	Censoring	Last day known to be live
Study termination for reasons other than those listed above (e.g., withdrawal of informed consent, major protocol deviation, etc.)	Censoring	Last day known to be live

Safety analysis

Safety analyses are based on ES unless otherwise specified.

Drug exposure

The actual duration of exposure, actual cumulative total dose, actual dose intensity (ADI), relative dose intensity (RDI), and duration of administration will be summarized for different investigational drugs, and a list of investigational drug use will be provided.

Actual cumulative total dose (mg): Cumulative total dose of investigational drug from start of first dose until last dose.

Actual duration of exposure is calculated as follows. Note: The ADI is calculated regardless of patient death or cut-off date.

- Actual duration of exposure $_{Tisle}$ (days) = MIN (date of last dose + 20, date of death, cut-off date) - date of first dose + 1
- Actual duration of exposure $_{Nab\text{-}P}$ (days) = MIN (date of last dose + 6, date of death, cut-off date) - date of first dose + 1
- Actual duration of exposure $_{Epiru}$ (days) = MIN (date of last dose + 20, date of death, cut-off date) - date of first dose + 1
- Actual duration of exposure $_{Cyclo}$ (days) = MIN (date of last dose + 20, date of death, cut-off date) - date of first dose + 1

ADI $_{Tisle}$ (mg/cycle) = 21*actual cumulative total dose $_{Tisle}$ /(date of last dose - date of first dose + 21)

ADI $_{Nab\text{-}P}$ (mg/cycle) = 21*actual cumulative total dose $_{Nab\text{-}P}$/(date of last dose - date of first dose + 7)

ADI_{Epiru} (mg/cycle) = 21*actual cumulative total dose $_{CBP}$/(date of last dose - date of first dose + 21)

ADI_{Cyclo} (mg/cycle) = 21*actual cumulative total dose $_{CBP}$/(date of last dose - date of first dose + 21)

PDI_{Tisle} = 200 mg/cycle

$PDI_{Nab\text{-}P}$ = Cumulative total planned dose of nab-paclitaxel (mg)/maximum number of dosing cycles

PDI_{Epiru} = Cumulative total planned dose of epirubicin (mg)/maximum number of dosing cycles

PDI_{Cyclo} = Cumulative total planned dose of cyclophosphamide (mg)/maximum number of dosing cycles

Relative dose intensity is calculated as follows: RDI (%) = 100%*ADI/PDI

Note:

The maximum number of dosing cycles planned does not exceed the maximum number of cycles actually administered.

The planned nab-paclitaxel dose is calculated as 100 (mg/m^2)* body surface area (m^2) using the "planned dose" in EDC "Use of nab-paclitaxel" page.

Adverse event

Drug therapy part

All AE records will be coded using MedDRA Chinese 24.0 or its latest version, and severity will be graded according to NCI CTCAE V5.0.

The following adverse event categories are summarized and listed:

- TRAEs during neoadjuvant therapy period
- ≥ Grade 3 TRAEs during neoadjuvant therapy period
- SAEs during neoadjuvant therapy period
- Study drug-related adverse events during neoadjuvant therapy period
- TRAEs leading to dose modification of investigational drug during neoadjuvant therapy period
- TRAEs leading to death during neoadjuvant therapy period

The number and incidence of TRAEs, SAEs, TRAEs related to treatment, and TRAEs leading to dose modification of investigational drug during neoadjuvant therapy period are summarized by system organ class SOC, preferred term PT, and severity.

Procedure section

Adverse events in the procedure section summarize those occurring during the surgical period.

All AE records are coded using MedDRA Chinese 24.0 or its latest version, and severity of procedure-related adverse events are graded according to Clavien-Dindo.

The following adverse event categories are summarized and listed:

- TRAEs during the surgical period
- TRAEs related to surgery during the surgical period
- ≥ Grade III TRAEs related to surgery during the surgical period
- SAEs related to surgery during the surgical period

- TRAE leading to death related to surgery during the surgical period

The number and incidence of TRAEs, SAEs, and TRAEs leading to death related to surgery are summarized by system organ class SOC, preferred term PT, and severity.

When summarizing the number and incidence of adverse events, if the same subject experiences the same adverse event several times, it shall be recorded as one time, and summarized according to the most severe CTCAE grade; when summarizing the number of adverse events, if the same subject experiences the same adverse event several times, it shall be recorded as multiple times, and each record in EDC data shall be counted as one case.

Laboratory test

Laboratory values at each time point are summarized as follows, including changes from baseline, as appropriate. Cross-tabulations of clinical significance test results at each time point before and after administration are listed, and a listing of all clinically significant abnormalities is provided (see Appendix 2 for test items):

- Hematology
- Clinical chemistry
- Clinical chemistry - Myocardial enzyme
- Urinalysis
- Coagulation function
- Thyroid function
- Viral load
- Pregnancy test
- Peripheral blood lymphocyte subsets

Vital signs and weight

Vital signs and weight (including changes from baseline) at each time point are summarized. Cross-tabulations of clinical significance test results at each time point before and after administration are listed, and a listing of all clinically significant abnormalities is provided. See Appendix 1 for test items.

ECOG performance status scores

ECOG scores at each time point are summarized, and cross-tabulations of scores at each time point before and after administration are listed. See Appendix 3 for test items.

12-lead ECG

12-Lead ECGs (including changes from baseline) at each time point are summarized. Cross-tabulations of clinical significance test results at each time point before and after administration are listed, and a listing of all clinically significant abnormalities is provided. See Appendix 1 for test items.

Echocardiogram/MUGA

Left ventricular ejection fraction (including change from baseline) at each time point is summarized. Cross-tabulations of clinical significance test results at each time point before and after administration are listed, and a listing of all clinically significant abnormalities is provided.

Interim analysis

Based on the group sequential design of the binary endpoint of single-arm trials, an interim efficacy and safety analysis is scheduled for the trial after enrollment of 48 patients. If there are ≤ 20 responders for pCR (ypT0/Tis ypN0), the trial will be terminated for ineffectiveness; if there are ≥ 30 responders for pCR, the primary endpoint is reached and the trial can be early terminated.

Exploratory analysis

Peripheral blood lymphocyte subpopulation count and ratio in peripheral blood at each time point could be summarized based on ES.

Based on ES, immune-related markers (expression levels of PD-L1 TC and IC) in tumor tissue at each time point could be summarized.

Stratified analysis of the primary efficacy endpoint is performed based on baseline PD-L1 status of subjects in the neoadjuvant therapy period based on ES.

Sample size consideration

Approximately 65 subjects will be recruited.

Based on the group sequential design of the binary endpoint of single-arm trials, an interim efficacy and safety analysis is scheduled for the trial after enrollment of 48 patients. If there are ≤ 20 responders for pCR (ypT0/Tis ypN0), the trial will be terminated for ineffectiveness. If there are ≥ 30 responders for pCR, the primary endpoint is reached and the trial can be early terminated. If the number of patients who achieved pCR was greater than or equal to 30, accrual could be terminated early after reaching the primary endpoint. This design yielded a one-sided type 1 error of 0.05 and a power of 80.0% when the true response rate was 56%. In this case, the trial can be early terminated as the primary endpoint is reached.

References

1. Simon R (1989). Controlled Clinical Trials 10: 1-10.
2. Brookmeyer, R., and Crowley, J. (1982). “A Confidence Interval for the Median Survival Time.” Biometrics 38:29–41.
3. Greenwood, M. (1926). The Natural duration of Cancer. Reports on Public Health and Medical subjects , 1-26.
4. FDA-Guidance-for-Industry-Clinical-Trial-Endpoints-for-the-Approval-of-Cancer-Drugs-and-Biologics_2007

Appendix 1 Schedule of Assessments

**Assessment items**	**Screening**[1]	**Neoadjuvant therapy period**[2]				**Safety follow-up**[3]	**Radical breast cancer surgery**	**Safety follow-up**[3]	**Early termination visit**[4]	**Long-term follow-up**[5]	**Survival follow-up**[6]
**Day (Time window)**	**-28 to -1**	**C1-4 D1 (±3)**	**C1-4 D8 (±3)**	**C1-4 D15 (±3)**	**C5-8 D1 (±3)**	**30 ± 7 days after last dose of investigational drug**		**30 ± 7 days after surgery**	**Within 30 days after last dose of investigational drug**	**Approximately every 3 months (± 14 days)**	
Informed consent[1]	x										
Inclusion/exclusion criteria	x										
Demographics/medical history	x										
Prior and concomitant medications[7]	x	x				x			x		
Complete physical examination[8]	x										
Vital signs, height, weight[9]	x	x	x			x		x	x	x	
ECOG score[10]	x	x	x			x		x	x	x	
Anti-cancer therapy after end of treatment										x	x
Tislelizumab administration		x			x						
Nab-paclitaxel administration		x	x	x							
Epirubicin administration					x						
Cyclophosphamide administration					x						
Radiotherapy (if applicable)											
AEs[11]	x	x				x		x	x		
12-lead ECG[12]	x					x	x		x		

Assessment items	Screening[1]	Neoadjuvant therapy period[2]				Safety follow-up[3]	Radical breast cancer surgery	Safety follow-up[3]	Early termination visit[4]	Long-term follow-up[5]	Survival follow-up[6]
**Day (Time window)**	**-28 to -1**	**C1-4 D1 (±3)**	**C1-4 D8 (±3)**	**C1-4 D15 (±3)**	**C5-8 D1 (±3)**	**30 ± 7 days after last dose of investigational drug**		**30 ± 7 days after surgery**	**Within 30 days after last dose of investigational drug**	**Approximately every 3 months (± 14 days)**	
Echocardiography or MUGA[12]	x					x	x		x		
Pregnancy test - Urine/serumβ -HCG[13]	x										
PT/INR and aPTT/PTT[14]	x					x	x		x		
Hematology[15,16]	x [16]	x	x	x	x	x	x	x	x	x	
Clinical biochemistry[15,16]	x [16]	x	x	x	x	x	x	x	x	x	
Urinalysis[6,16]	x [16]	C2 D1				x	x	x	x	x	
T3, FT4 and TSH[15,16]	x[16]	C4 D1				x	x	x	x	x	
HBV/HCV test[17]		As clinically indicated									
Breast MRI[18]	x	C3, C5, C7 D1					x			As clinically indicated	
Analysis of blood and tissue biomarkers[19]	x								x		
Tumor tissue biopsy for translational research[20]	x	C2 D1					x				
Radical breast cancer surgery							x				
Baseline tumor assessment	x										
Disease progression assessment[21]		C3, C5, C7 D1				x		x	x	x	x
pCR assessment							x				
FFPE tissue or TNBC status slides[22]	x	C2 D1					x				

Abbreviations: AEs = adverse events; ECG = electrocardiogram; FFPE = formalin-fixed paraffin-embedded; FT4 = free thyroxine; HCG = human chorionic

gonadotropin; MRI = magnetic resonance imaging; MUGA = cardiac radionuclide scan; PT/INR = prothrombin time/international normalized ratio; aPTT/PTT = activated prothrombin time/partial thromboplastin time; T3 = triiodothyronine; TNBC = triple-negative breast cancer; TSH = thyroid-stimulating hormone;

1. Patients must sign a written informed consent form prior to any study-specific tests or procedures during the screening period. Standard of care test or test results prior to obtaining informed consent and within 28 days prior to the first dose of investigational drug may be used for screening assessments without repeating the same test.
2. In general, assessments/procedures should be performed every 6 weeks (± 7 days) during treatment period unless otherwise specified. Each treatment cycle consists of 3 weeks (21 days). If treatment is delayed, all procedures should be performed according to the new dosing schedule.
3. Safety follow-up: defined as from signing of informed consent to the date of last AE data collection. AEs collected include: 1) chemotherapy-related AEs until 30 ± 7 days after the last dose; 2) immune-drug-related AEs until 90 ± 7 days after the last dose of tislelizumab; 3) surgery-related AEs until 30 ± 7 days after the surgery.
4. If a subject discontinues all protocol-specified therapies after Cycle 1 treatment through the surgery, the subject should return to the site for early discontinuation visit within 30 days after the last dose of investigational drug or before the start of new anticancer therapy, whichever occurs first. If routine laboratory tests (e.g., hematology, clinical chemistry) are performed within 7 days prior to early termination visit, they are not need to be repeated. If the previous tumor assessment is performed within 6 weeks prior to early termination visit, it does not need to be repeated at the early termination visit. If investigational drug is originally interrupted due to AE and then permanently discontinued, the early termination visit can have followed, but no later than the allowable time of dose delay + 7 days.
5. In-office long-term follow-up assessment is performed every 3 months (± 1 month) after surgery to assess recurrent or metastatic disease. Other tests/imaging assessments (e.g., bone/liver scan) for recurrent or metastatic disease will be performed at the discretion of the subject's treating physician according to local standard of care or at the onset of symptoms. Unless a patient requests withdrawal from follow-up, all patients will be followed by the site for survival status and subsequent anti-cancer therapy. Long-term follow-up ends at 3 years after initiation of drug therapy.
6. If a subject is withdrawn from treatment due to recurrent or metastatic disease at any time during the neoadjuvant therapy, or long-term follow-up periods, survival follow-up information should be collected by telephone, patient medical records, and/or clinical visits every 6 months (± 1 month) until death, loss to follow-up, withdrawal of informed consent, or study termination by the sponsor, whichever occurs first.
7. Prior medications, record all medications administered within 30 days prior to the screening visit. Concomitant medications, record new medications that start during the screening period to the safety follow-up visit after the surgery period or early discontinuation (whichever occurs first). Record all medications for the treatment of AE.
8. Physical examination includes: 1) head, eyes, ears, nose, throat; 2) cardiovascular; 3) dermatologic; 4) musculoskeletal; 5) respiratory; 6) gastrointestinal; and 7) neurological systems.

9. Vital signs include body temperature, pulse rate, respiratory rate and blood pressure. Height will be measured at screening only; weight will be measured at Baseline and at each cycle. Vital signs are collected during the treatment cycles.
10. ECOG performance status test is performed within 10 days prior to the first dose of trial treatment. ECOG performance status will also be scored prior to each investigational drug administration, at safety follow-up, at the early termination visit, and at the long-term follow-up visit in each treatment cycle.
11. AEs and laboratory safety measures will be graded according to NCI CTCAE v5.0, and all AEs, whether graded according to CTCAE or not, will also be evaluated for severity.
12. ECG and MUGA/ECHO should be performed at screening, safety visit, and early termination visit. ECG and MUGA/ECHO do not need to be repeated if performed within 3 days prior to first dose of investigational drug and within 3 days prior to visit.
13. For women of childbearing potential, serum or urine pregnancy test should be performed within 72 hours prior to the first dose of study treatment. If urine pregnancy is positive or cannot be confirmed negative, serum pregnancy test will be required at the local site laboratory.
14. For subjects receiving anticoagulant therapy, coagulation factors (PT/INR and aPTT/PTT) should be measured at baseline and closely monitored during treatment period and safety follow-up.
15. Hematology, clinical chemistry, urinalysis, and thyroid panel: If the screening laboratory tests are not performed within 72 hours prior to investigational drug administration on Cycle 1 Day 1, they should have been repeated and results reviewed prior to investigational drug administration.
16. Unresolved laboratory abnormality belonging to drug-related AEs should be followed until resolution. If the laboratory test results are within normal limits, the laboratory test does not need to be repeated after the end of treatment.
17. HBV/HCV test: The test will be performed by local site at screening and will include HBV/HCV serology (HBsAg, HBsAb, HBcAb, and HCV antibody) and viral load assessment (HBV DNA and HCV RNA). Patients must be tested at screening. In addition, for patients with detectable HBV DNA at screening, corresponding viral load test will be performed every 4 cycles (i.e., cycle 5 day 1 of neoadjuvant therapy period) since Cycle 5.
18. Breast MRI is optional.
19. Analysis of blood and tissue biomarkers: including PD-L1 testing, cyTOF, WES, olink, TCR, scRNA, etc.
20. Optional core needle biopsy will be performed only for subjects willing to participate in the study.
21. Assessments include (according to local or institutional guidelines): disease progression precluding radical breast cancer surgery, local or distant recurrence, development of second primary malignancy, or death. Results will be recorded in the electronic data capture (EDC) system.

22. Formalin-fixed paraffin-embedded (FFPE) tumor tissue samples or slides obtained by the subject at the initial diagnosis may be submitted to the designated central laboratory at screening for confirmation of the subject's TNBC status provided that no new biopsy samples are available due to site inaccessibility or medical contraindications and that consent is obtained from the sponsor.

Appendix 2 Clinical Laboratory Assessment

Clinical chemistry	Hematology	Urinalysis	Peripheral blood lymphocyte subsets	Others
Alkaline phosphatase	Hematocrit	Glucose	CD3+(%)T cells	β-HCG[a]
Alanine aminotransferase	Hemoglobin	Protein	CD19+(%)B cells	PT (INR)[b]
Aspartate aminotransferase	Platelet count	Blood	CD3+CD4+ %	aPTT/PTT[b]
Albumin	WBC count	Urine specific gravity	CD3+CD8+ %	T3[c]
Total bilirubin	Red blood cell count	Urine pregnancy test	CD16+CD56+ %(NK)	FT4
Direct bilirubin	Neutrophil count			TSH
Blood urea nitrogen or urea[d]	Lymphocytes count		Total lymphocytes (T+B+NK)	
Uric acid			CD4/8 cell ratio (Th/Ts)	
Kalium			Absolute count of CD3+ cells	
Natrium			Absolute count of CD19+ cells	
Corrected calcium[e]				
Phosphorus			Absolute count of CD3+CD4+ cells	
Creatinine or creatinine clearance			Absolute count of CD3+CD8+ cells	
Glucose			Absolute count of CD16+CD56+ cells	
Lactate dehydrogenase			Absolute count of CD45+ cells	
Total protein				
CK[f]				
CK-MB[f,g]				

Abbreviations: CK = creatine kinase; CK-MB = creatine kinase isoenzyme; WBC = white blood cell;

a. Performed for females of childbearing potential only. Urine pregnancy test is preferred. Serum pregnancy test is required if the urine test is positive or cannot be confirmed negative. Serum or urine pregnancy test should be performed within 72 hours prior to the first dose of study treatment.

b. Coagulation factors (PT/INR and aPTT/PTT) should be tested as part of the screening procedure according to the time points specified in Appendix 1. For subjects receiving anticoagulant therapy, additional tests are performed as clinically indicated.

c. Total T3 is preferred; free T3 can be detected if total T3 not available.

d. Blood urea nitrogen is preferred; urea may be tested if BUN not available.

e. If corrected calcium testing is not feasible at the local laboratory, total calcium determination may be performed instead.

f. Patients receiving tislelizumab will undergo creatine kinase (CK) and creatine kinase cardiac isoenzyme (CK-MB) testing. Creatine kinase (CK, an enzyme produced by the heart muscle) and CK-MB tests (replaced with troponin I and/or troponin T if CK-MB monitoring is not available) are performed on Day 1 of each dosing cycle to confirm that neither drug causes damage to your heart. CK and CK-MB testing are no longer required if tislelizumab has been permanently discontinued.

g. If CK-MB is not detected, please assess troponin I and/or troponin T.

Appendix 3 ECOG performance status scores

Grading	Description
0	Fully active, able to carry on all predisease activities without restriction
1	Restricted in strenuous activity but ambulatory and able to carry out work of a light or sedentary nature, e.g., light house work, office work
2	Capable of all self-care, but no work activities. Up and about more than 50% of waking hours
3	Capable of only limited self-care, confined to bed or chair more than 50% of waking hours
4	Completely disabled. Cannot carry on any self-care. Totally confined to bed or chair
5	Death

See (Oken et al 1982) Eastern Cooperative Oncology Group, Robert Comis M.D., Group Chair for details.

Appendix 4 Judgment Rules for Concomitant Medication

If the start/end date of prior and concomitant medication is incomplete, the type of concomitant medication should be judged by the available information as far as possible:

1) It is prior medication if end date is earlier than the date of first dose; concomitant medication if end date is later than the date of first dose
2) If not judged based on end date, it is defined as concomitant medication

Appendix 5 Judgment Rules for TRAEs

If the start/end date of adverse event is incomplete, the TRAE should be judged based on the available information as far as possible:

1) It is not TRAE if start date is earlier than the date of first dose; TRAE if start date is later than the date of first dose
2) If not judged based on the start date, judged based on the end date: if the end date is earlier than the date of first dose, it is not a TRAE; if it is later than the date of first dose, it is a TRAE; and if not judged based on the end date, it is defined as a TRAE.

Appendix 6 Imputation Rules for Date of Death

If the date of death is incomplete, imputation rules are:

1) Date of death is YYY-MM

    a) If the date of death is in same month as the date of last follow-up, impute the subject's last day known to be live + 1.

    b) If later than the month of last day known to be live, impute first day of that month.

    c) No imputation is made if earlier than the month of last day known to be live (data error).

2) Date of death is YYYY

    a) Data are not imputed and censored date is defined as the last day known to be live of the subject.

    Note: The last day known to be live is defined in Appendix 7.

Appendix 7 Last Day Known to be Live

The last day known to be live is defined as the latest of the following dates and specifies the conditions under which this date is used as the last day known to be live.

Date name	Conditions of use
Date of administration	Actual dose administered is not null
Blood/urine sample collection date	No condition
Date of imaging	No condition
Start date of AE	AE name is not null
Start date of concomitant medication	Drug name is not null
